# Cannabinoid CB_2_ receptor drives trastuzumab resistance and predicts durable anti-HER2 response

**DOI:** 10.1038/s41388-026-03814-9

**Published:** 2026-05-11

**Authors:** Marta Seijo-Vila, Sofía A. Balsinde, Sandra Blasco-Benito, Isabel Tundidor, María Rubert-Hernández, Ana Montero-Calle, Rodrigo Barderas, Laura E. Kilpatrick, Simon Platt, Noemi Karsai, Isabel Philps, Olga M. Antón, Déborah Gómez-Domínguez, Ignacio Pérez de Castro, Carmen González-Lois, Diego García-Fresnadillo, Gala Silvestre-Egea, Antonio J. Sánchez-López, Esther Ramírez-Medina, María Catalina Rivas Prieto, Belén Almoguera Pérez-Cejuela, Luis Manso, Sandra Zazo, Noemí López-Ejeda, Francisco Palomino-Duque, María Turienzo-Durán, Nuria G. Martínez-Illescas, María Salazar-Roa, Sonia Castillo-Lluva, Stephen J. Hill, Manuel Guzmán, Eduardo Pérez-Gómez, Cristina Sánchez

**Affiliations:** 1 Department of Biochemistry and Molecular Biology, Faculty of Biological Sciences, Complutense University of Madrid, Madrid, Spain; 2 Instituto de Investigación Sanitaria Hospital 12 de Octubre (imas12), Madrid, Spain; 3 Hospital del Mar Research Institute (IMIM), Barcelona, Spain; 4 Chronic Disease Programme (UFIEC), Instituto de Salud Carlos III, Madrid, Spain; 5 CIBER Frailty and Healthy Aging (CIBERFES), Madrid, Spain; 6 Centre of Membrane Proteins and Receptors (COMPARE), University of Birmingham and University of Nottingham, The Midlands, Nottingham, UK; 7 Division of Biomolecular Science and Medicinal Chemistry, School of Pharmacy, Biodiscovery Institute, University of Nottingham, Nottingham, UK; 8 Division of Physiology, Pharmacology and Neuroscience, School of Life Sciences, University of Nottingham, Nottingham, UK; 9 Instituto de Investigaciones Sanitarias San Carlos (IdISSC), Madrid, Spain; 10 Gene Therapy Unit, Instituto de Investigación de Enfermedades Raras y Departamento de Desarrollo de Medicamentos y Terapias Avanzadas, Instituto de Salud Carlos III, Madrid, Spain; 11 Department of Pathology, Hospital Universitario Puerta de Hierro Majadahonda, Madrid, Spain; 12 Neuroimmunology Unit, Instituto de Investigación Sanitaria Puerta de Hierro-Segovia de Arana, Madrid, Spain; 13 Biobank, Instituto de Investigación Sanitaria Puerta de Hierro-Segovia de Arana, Madrid, Spain; 14 Department of Gynecology, Hospital Universitario Puerta de Hierro Majadahonda, Madrid, Spain; 15 Department of Medical Oncology, Hospital Universitario 12 de Octubre, Madrid, Spain; 16 Pathology Department, IIS-Hospital Universitario Fundación Jiménez Díaz-CIBERONC, Madrid, Spain; 17 Department of Biodiversity, Ecology and Evolution, Faculty of Biological Sciences, Nutritional Epidemiology (EPINUT) Research Group, Complutense University of Madrid, Madrid, Spain; 18 Instituto Ramón y Cajal de Investigación Sanitaria (IRYCIS), Instituto Universitario de Investigación en Neuroquímica (IUIN), and Centro de Investigación Biomédica en Red de Enfermedades Neurodegenerativas (CIBERNED), Madrid, Spain; 19 Present Address: Department of Gynecology, HM Montepríncipe University Hospital, Madrid, Spain

**Keywords:** Breast cancer, Predictive markers

## Abstract

Acquired or innate lack of response to standard HER2-targeted therapies remains a clinical issue in patients with HER2-positive breast cancer. Here, we investigated the role of the cannabinoid CB_2_ receptor (CB_2_R) in trastuzumab resistance. In human breast cancer samples, a decreased expression of HER2-CB_2_R heterodimers following neoadjuvant treatment, due to CB_2_R downregulation, was linked to poor long-term outcomes. Using various preclinical models, we demonstrate that CB_2_R drives trastuzumab resistance. Mechanistically, CB_2_R loss enabled cancer cells to evade antitumor IFN-γ signaling while promoting a shift from HER2-CB_2_R to HER2-EGFR heterodimers, thus reducing dependence on HER2 and increasing reliance on EGFR-mediated pathways. Moreover, EGFR inhibition restored trastuzumab sensitivity. In summary, we reveal an unprecedented role for CB_2_R as a key regulator of oncogenic and immune signaling in response to anti-HER2 therapy and its potential as a predictive biomarker of therapeutic efficacy. We also propose dual HER2/EGFR targeting and non-CB_2_R-selective cannabinoid therapies as potential strategies to overcome CB_2_R-mediated trastuzumab resistance. Together, these findings position the endocannabinoid system as a pivotal and actionable node to elucidate, anticipate, and counteract resistance to HER2-targeted therapies.

## Introduction

Breast cancer (BC) is the leading cause of cancer-related death in women [1]. One of the main subtypes of BC is characterized by the overexpression of HER2. This oncogene is a receptor tyrosine kinase that belongs to the HER family, which consists of four members: HER1 (EGFR), HER2, HER3, and HER4. Activation of these receptors occurs upon homo- or heterodimerization, resulting in the activation of pro-oncogenic signaling pathways that drive tumor progression [2]. HER2-positive (HER2 + ) BC management has experienced a significant improvement over the past two decades owing to the development of HER2-targeted therapies. Trastuzumab, a humanized monoclonal antibody that binds to the extracellular domain IV of HER2, was the first approved HER2-targeted therapy and remains the cornerstone of all HER2 + BC treatments [3]. The main mechanisms underlying trastuzumab antitumor action include HER2 internalization, the suppression of HER2-driven pro-oncogenic signaling, and the induction of antibody-dependent cell-mediated cytotoxicity (ADCC) [3]. The latter response involves immune cells, primarily natural killer (NK) cells, which are recruited to kill target HER2-overexpressing cells coated with trastuzumab [4].

The standard treatment course for most HER2 + BC patients begins with an initial preoperative (neoadjuvant) therapy (NAT), followed by an adjuvant treatment. NAT aims to evaluate treatment efficacy to determine the subsequent adjuvant treatment plan, as well as to reduce the extent of surgical intervention needed. Most NAT regimes involve dual HER2 blockade with trastuzumab and pertuzumab (an anti-HER2 antibody targeting the extracellular domain II of the receptor) in combination with chemotherapy. After NAT, patients who achieve pathological complete response (pCR) or have residual disease receive adjuvant treatments that also include trastuzumab [5–7]. Despite these advances, and although early HER2 + BC patients respond successfully to therapy in most cases, the metastatic stage is usually accompanied by treatment resistance and disease progression. Resistance mechanisms include restrictions for trastuzumab binding to HER2, aberrant activation of HER2 downstream signaling pathways, ADCC escape, and a switch to alternative pro-oncogenic drivers [8, 9]. This scenario underscores the need for a deeper understanding of the molecular mechanisms responsible for treatment resistance, as well as for the identification of predictive biomarkers that would enable early detection of which precise patients will respond to specific therapies, thus allowing for personalized treatment selection.

Recent studies have highlighted the potential role of the endocannabinoid system (ECS) in HER2 + BC progression. This cell communication system comprises two cannabinoid-sensing G protein-coupled receptors (CB_1_R and CB_2_R), their endogenous ligands (known as endocannabinoids), and the enzymes responsible for endocannabinoid synthesis and degradation [10]. In the context of BC, previous evidence supports that the pro-oncogenic activity of HER2 is regulated by CB_2_R. Specifically, the formation of HER2-CB_2_R heterodimers promotes HER2 signaling, while disruption of these protein complexes triggers antitumor responses [11, 12]. Hence, given the relevance of HER2-CB_2_R heterodimers in HER2 + BC physiopathology, this study aims to explore their potential involvement in the resistance to HER2-targeted therapies, particularly those based on trastuzumab.

## Materials and methods

### Human samples

All studies involving human samples were performed in accordance with the relevant guidelines and regulations, and approved by the respective hospital ethics committees (CEIm # 23/213 and 20/615, Hospital 12 de Octubre and Hospital Puerta de Hierro, respectively), with patients providing informed consent without compensation.

Two tissue microarrays (TMAs) were constructed using 1 mm cores from paraffin-embedded tissue blocks, arranged into a positionally encoded recipient paraffin block. The samples consisted of tumor biopsies from HER2+ cancer patients collected before and after neoadjuvant treatment (NAT), which included trastuzumab (± additional anti-HER2 therapies and/or chemotherapy). **TMA H12O** comprised 37 samples from patients treated at Hospital 12 de Octubre (Madrid, Spain) between 1999 and 2013, including 31 paired samples (pre- and post-NAT from the same patient). **TMA HPH** included 42 paired duplicate samples from patients treated at Hospital Puerta de Hierro (Madrid, Spain) between 2012 and 2022 and deposited at the hospital’s biobank (Carlos III Health Institute Biomodels and Biobanks Platform – PT23/00015).

### Cell lines and cultures

Cell lines were obtained from commercial vendors whenever available. Cell lines not directly purchased from certified suppliers were authenticated by short tandem repeat profiling at the Genomics Core Facility of the Instituto de Investigaciones Biomédicas Alberto Sols (Madrid, Spain). All cell lines were cultured at 37 °C in a humidified atmosphere with 5% CO₂ and tested weekly for *Mycoplasma* contamination with PuReTaq Ready-To-Go PCR Beads (#27-9557-02, GE Healthcare, Madrid, Spain) and the following primers: P1, 5´ GGC GAA TGG GTG AGT AAC ACG 3´and P2, CGG ATA ACG CTT GCG ACC TAT G 3. After thawing, cell lines were used for a maximum of 25 passages. Human HER2+ female breast cancer cell lines **BT474 TS** (trastuzumab-sensitive, #HTB-20) and **TR** (trastuzumab-resistant, #CRL-3247) were obtained from the American Type Culture Collection (ATCC, Manassas, VI, USA) in 2021 and 2022, respectively.

The CB_2_R knockout (**KO**) cell line was generated by knocking out CB_2_R expression in TS cells using CRISPR/Cas9. The following RNA guide targeting CB_2_R exon 3 was cloned into the pLv-puro-sgRNA lentiviral plasmid: TCC GGA ATC ATC TAC ACC TA TGG (Addgene #7140, Watertown, MA, USA). TS cells stably expressing Cas9 (via infection with a pCLIP-hCMV-Cas9-Nuclease-Blast lentiviral plasmid, kindly donated by Dr. Markus Müschen, City of Hope Comprehensive Cancer Center, Duarte, CA, USA) were infected with the CB_2_R-targeting guide. Cells incorporating both Cas9 and guide were selected via antibiotic resistance, and CB_2_R knockout was confirmed by genomic PCR.

The scramble control (**SCR**) cell line was generated using the same protocol but with a scramble guide (CCT AAG GTT AAG TCG CCC TCG CTC GAG CGA GGG CGA CTT AAC CT TAGG). The CB_2_R rescue (**RESC**) and **TR CB**_**2**_**R** cell lines were derived from CB_2_R KO and TR cells, respectively, via lentiviral transduction of the pLv-neo-CMV-hCNR2 plasmid. Briefly, lentiviral particles were produced by transfecting HEK293T cells with envelope (pMD2.G, Addgene #12259), packaging (psPAX2, Addgene #12260), and donor plasmids (CB_2_R, pLv-neo-hCNR2, Vector Builder, Neu-Isenburg, Germany). Conditioned medium containing recombinant lentiviruses was collected, filtered, and used to infect CB_2_R KO and TR cells.

Trastuzumab sensitive/resistant cells derived from **PDX118** (a patient-derived xenograft established from a HER2⁺ breast tumor) were kindly provided by Dr. Enrique Arenas (Institut de Recerca Contra la Leucèmia Josep Carreras. Barcelona, Spain) and are described in [13].

**CHO-K1** (#CCL-61, RRID:CVCL_0214) and **HEK293T** cells (#CRL-3216, RRID:CVCL_0063) (both female) were obtained from EACR (Nottingham, UK) and ATCC, respectively. The **HEK HER2** and **HEK CB**_**2**_**R** cell lines were generated by lentiviral transduction of the pLv-Bsd-CMV-hERBB2 and pLv225-puro-HA-hCNR2 plasmids (Vector Builder), respectively, following the protocol described above. **HEK HER2-CB**_**2**_**R** cells were generated by simultaneous infection with both plasmids.

The NK cell line **NK92-CD16-GFP** (male) was also obtained from ATCC in 2016 (#CRL-2408, RRID:CVCL_3755).

All BT474-derived and PDX-derived cell lines were cultured in DMEM/F12 supplemented with FBS, and penicillin/streptomycin. HEK293T cells were grown in DMEM supplemented with FBS, ultraglutamine, and penicillin/streptomycin. Cells used for displacement experiments were grown in DMEM/F12 (CHO-K1) or DMEM (HEK293T) supplemented just with FBS. NK92-CD16-GFP cells were cultured in RPMI supplemented with FBS, human serum, IL-2, glutamine, sodium pyruvate, and β-mercaptoethanol.

### Cell viability assays

Cells were seeded at semi-confluence and cultured for 24 h. Viability was then assessed using crystal violet staining. Briefly, cultures were incubated with 0.1% crystal violet solution in methanol/water for 20 min. After several washes, the resulting crystals were dissolved in methanol, and absorbance was measured at 570 nm.

When cells were treated with trastuzumab, erlotinib, or cannabinoids alone, their viability was measured after 5, 3, and 1 days of drug exposure, respectively. For experiments with combined treatments, cell viability was analyzed 4 days after drug challenge. Cannabinoids used were HU308 (CB2R-selective agonist, Tocris Bioscience #3088, Bristol, UK), Δ^9^-tetrahydrocannabinol (THC, a CB_1_R/CB_2_R mixed agonist, THC Pharm Gmbh #THC-1099, Frankfurt, Germany) and three *Cannabis sativa* extracts: two of them rich in THC (Cannabis Extracts 1 and 2, CE1 and CE2), and one with a balanced THC:CBD ratio (CE3). CE1 was kindly donated by Aunt Zelda’s (California, USA), and C2 and C3 by Curativa Group (Colombia). The composition of the three extracts is shown in Supplementary Table S2.

In tumor cell + NK cell co-culture experiments, tumor cell lines were seeded at semi-confluence in complete medium and incubated with trastuzumab for 1 h. Subsequently, NK92-CD16-GFP cells were added at a 1:4 ratio (tumor:NK cells) in NK cell medium lacking IL-2, and cell viability was assessed 24 h later.

### Proximity ligation assays (PLAs)

Receptor heteromers were detected using the Duolink In Situ PLA Detection Kit (Sigma-Aldrich, St. Louis, MA, USA) according to the manufacturer’s instructions. For TMAs and animal-derived xenograft sections, samples were deparaffinized, subjected to heat-induced antigen retrieval in sodium citrate buffer, and permeabilized with 0.05% Triton X-100. For cell cultures, cells were seeded on glass coverslips, challenged with the corresponding treatment when indicated, fixed with paraformaldehyde, and permeabilized with Triton X-100. Samples were then incubated with primary antibodies: rabbit anti-CB_2_R (Cayman Chemical, #101550-1, RRID:AB_327841), mouse anti-HER2 (Santa Cruz Biotechnology, #sc-08, RRID:AB_627998), rabbit anti-EGFR, and rabbit anti-IFNGR1 (Cell Signaling Technology, #4267, RRID:AB_2246311, and #34808, RRID:AB_2799061, respectively). Secondary antibodies conjugated to PLUS and MINUS complementary nucleotide probes (anti-rabbit PLUS and anti-mouse MINUS; Sigma-Aldrich #DUO92002 and #DUO92004, respectively) were added, followed by ligase and polymerase in the presence of red fluorescent nucleotides. Nuclei were stained with DAPI. Samples were analyzed via confocal microscopy and processed with ImageJ software. Heteromer expression was quantified as the number of red fluorescent dots (indicating receptor proximity sufficient for probe complementation, circularization, and amplification) per total cells in the field.

Kaplan-Meier curves for disease-free survival based on heteromer expression were generated using the Kaplan-Meier Plotter (KM plotter) online tool. The best threshold cutoffs were automatically determined by the software.

### Immunohistochemistry assays

Paraffin-embedded tissue sections were subjected to a heat-induced antigen retrieval and subsequent incubation with either rabbit anti-HER2 (Herceptest, 1:500, Agilent Dako, Santa Clara, CA, USA), rabbit anti-CB_2_R (Cayman Chemical, #101550-1, RRID:AB_327841, 1:2500), or rabbit anti-EGFR (Leica Biosystems, #NCL-EGFR, RRID:AB_442085, or Cell Signaling Technology, #4267, RRID:AB_2246311) antibodies.

Immunodetection was performed using the Envision method with diaminobenzidine as the chromogen. CB_2_R and EGFR expression were quantified in the TMAs assigning scores to each sample [0 (no staining), 1 (weak staining), 2 (moderate staining), or 3 (high staining)]. HER2 staining was scored in accordance with the HercepTest manufacturer’s guidelines.

Kaplan-Meier plots for disease-free survival based on HER2, CB_2_R, or EGFR expression were generated as explained in the PLA methods section.

### Real-time quantitative PCR (qPCR)

RNA extraction was performed using the NucleoZOL kit (#740404, Macherey-Nagel, Dueren, Germany) following the manufacturer’s instructions. Reverse transcription to cDNA was carried out using the Transcriptor First Strand cDNA Synthesis Kit (#4897030001, Roche Life Science, Barcelona, Spain). qPCR was performed with the following primers and SYBR Green probe (Roche Life Science #4913914001): *CNR2*: sense CAC TGA TCC CCA ATG ACT ACC T, antisense CAT GCC CAT AGG TGT AGA TG; *ERBB2*: sense GGG AAA CCT GGA ACT CAC CT, antisense CCC TGC ACC TCC TGG ATA; *ERBB1*: sense ACA CAG AAT CTA TAC CCA CCA GAG T, antisense ATC AAC TCC CAA ACG GTC AC; *ERBB3*: sense TGA ATG GCC TGA GTG TGA CC, antisense CCC CTG ACA GAA TCT CGG TG; *ERBB4*: sense GAG CAA GAA TTG ACT CGA ATA GG, antisense TTC CTG ACA TGG GGG TGT AG; *IFNGR1*: sense CCT TGT CAT GCA GGG TGT GA, antisense TTG GTG TAG GCA CTG AGG AC; *JAK1*: sense GGC TTC TGA GAC ACA CGC TT, antisense CAG CTG TCC AGT GTT CTC CA; *JAK2*: sense GCC GGG TTT CAG AAG CAG G, antisense TTC AGA ACA TTT GCC GTC GC; *STAT1*: sense GCT CGT TTG TGG TGG AAA GAC, antisense TCT CTC ATT CAC ATC TCT CAA CTT; *ACTB*: sense CCA ACC GCG AGA AGAT GA, antisense CCA GAG GCG TAC AGG GAT AG; *TBP*: sense CCC ATG ACT CCC ATG ACC, antisense TTT ACA ACC AAG ATT CAC TGT GG. Gene expression was calculated using the ΔΔCt method and normalized to reference genes TBP (TATA-binding protein) and ACTB (β-Actin).

### RNA sequencing (RNAseq)

RNA from cell cultures was isolated as described above. Concentration and RNA integrity (>7) were determined, and 1 μg per sample was utilized for RNA-seq. Sequencing and results analysis (with Genome One software) were performed by Dreamgenics S.L. (Oviedo, Spain). The generated datasets are available through the Gene Expression Omnibus repository under the accession number GSE300749. Gene expression level was represented as a colored cell, with a color key interpretation indicated where appropriate. The identification of the biological processes modulated by the differentially expressed genes was performed by using the pathfindR tool and the repositories of Kyoto Encyclopedia of Genes and Genomes (KEGG) and Gene Ontology (GO), and GSEA (Gene Set Enrichment Analysis).

### Proteomic analyses

Cultured cells were lysed in GST lysis buffer [10% glycerol, 100 mM NaCl, 2 mM MgCl₂, 50 mM Tris-HCl pH 7.4, 1% tergitol (Sigma-Aldrich, #NP40S)] supplemented with protease and phosphatase inhibitors. Samples were centrifuged, and protein concentration in supernatants was determined using the Bradford method. Total protein was split into two fractions: one for validating target protein expression (input) and another (IP) to proceed with a protein co-immunoprecipitation (co-IP), that started with an incubation with G-Sepharose beads (or anti-HA antibody-bound beads for HEK293T HA-CB_2_R cells). After bead incubation, beads were removed, and samples were incubated with primary antibodies. G-Sepharose beads were then reintroduced, and target proteins were co-immunoprecipitated via centrifugation. Proteins were then eluted by boiling in Laemmli loading buffer. Analysis of the co-IP procedure was performed by Western blot (see below).

Following co-IP, proteins were separated by SDS-PAGE and stained with Coomassie Brilliant Blue G-250 to confirm proper protein separation. Gel lanes were excised, destained, dehydrated, and subjected to reduction/alkylation. After rehydration, proteins were digested with trypsin, and resulting peptides were extracted, purified, and analyzed by mass spectrometry.

LC-MS/MS parameters followed the protocol by Montero-Calle et al. [14]. Peptides were separated using a nano Easy-nLC 1000 system (Thermo Fisher Scientific, Madrid, Spain) with an Acclaim PepMap 100 precolumn (Thermo Fisher Scientific #164946) and an RSLC PepMap C18 column (Thermo Fisher Scientific #11362013). Mobile phase flow rate was 300 nL/min, with 0.1% formic acid (FA) in water (buffer A) and 0.1% FA in 100% acetonitrile (buffer B) for peptide elution over a 102-min gradient. MS/MS analysis was performed on a Q Exactive using data-dependent acquisition, selecting the top 15 most intense precursor ions for fragmentation after each scan.

Mass spectrometry data were analyzed with Proteome Discoverer (v1.4.1.14, Thermo Fisher Scientific). Raw spectra files were searched against the *Swiss-Prot_2016_10.fasta* database (Homo sapiens, 20121 protein sequences) using Mascot (v2.6, Matrix Science). Precursor and fragment mass tolerances were set to 10 ppm and 0.02 Da, respectively. Parameters included two missed cleavages, fixed carbamidomethylation of cysteines, and variable methionine oxidation/N-terminal acetylation. Peptides were filtered using Percolator (q-value threshold: 0.01). Venn diagrams were generated using the jvenn web tool. The Reactome Pathway Database was used to identify signaling pathways associated with potential interactors.

### Western blot

Cells were scraped into DDM buffer (or RIPA buffer for JAK/STAT protein analysis) supplemented with protease and phosphatase inhibitors. Protein extracts were separated by SDS-PAGE and transferred to PVDF membranes using a Trans-Blot® SD Semi-Dry Transfer Cell (Bio-Rad, Madrid, Spain). Membranes were then incubated with the appropriate primary antibodies: mouse anti-EGFR (4267, RRID:AB_2246311), rabbit anti-JAK1 (3344, RRID:AB_2265054), rabbit anti-JAK2 (3230, RRID:AB_2128522), rabbit anti-p-STAT1 (9167, RRID:AB_561284), rabbit anti-STAT1 (9172, RRID:AB_2198300), rabbit anti-IRF1 (8478, RRID:AB_10949108), all from Cell Signaling Technology; rabbit anti-CB_2_R (SAB1306696), mouse anti-Vinculin (V9264, RRID:AB_10603627), and mouse anti-β Actin (A5441, RRID:AB_476744), all from Sigma-Aldrich. After several washes, membranes were incubated with the corresponding HRP-conjugated secondary antibodies, and the signal was developed using a self-prepared ECL reagent. The bioluminescence was captured with the ImageQuant LAS 500 system (GE HealthCare) and the densitometric analysis was performed using Image LabTM software (Bio-Rad).

### Biotinylation assays

HER2 expression specifically at the plasma membrane was assessed by protein biotinylation assays. Following an initial 15 min incubation on ice, cells were exposed to sulfobiotin for 30 min in the dark. Cells were then washed with L-lysine and lysed in DDM buffer supplemented with protease and phosphatase inhibitors, and protein concentration was determined by Bradford assay.

A portion of each sample was reserved to assess the expression of proteins of interest (input), while the remainder was incubated with streptavidin beads to capture biotinylated proteins. Samples were centrifuged, washed with DDM buffer lacking inhibitors, and prepared in Laemmli loading buffer. After denaturation they were subjected to SDS-PAGE and transferred to PVDF membranes, that were processed as described in the Western blot section. Results were normalized using sodium-potassium ATPase and a loading control as references.

### Flow cytometry assays

Trastuzumab-HER2 binding was analyzed by flow cytometry using fluorescein isothiocyanate-conjugated trastuzumab (trastuzumab-FITC; #10-2002-F, Abeomics, San Diego, CA, USA). Briefly, cells were detached from culture plates using TripLE™ Express (#12-605-010, Gibco, Eindhoven, The Netherlands) and transferred to 96-well V-bottom plates in staining buffer (PBS containing FBS, BSA, and sodium azide). Plates were centrifuged and pellets were incubated with trastuzumab-FITC for 30 min in the dark. Samples were then transferred to round-bottom polystyrene tubes and stained with 7-aminoactinomycin D (7-AAD) as a viability marker 15 min prior to analysis. Flow cytometry was performed on a FACSCalibur system (Becton Dickinson, Franklin Lakes, NJ, USA), with data processed using FlowJo™ software (BD Biosciences, San Jose, CA, USA).

### Trastuzumab affinity displacement assays with NanoBiT

HiBiT-EGFR was a gift from Promega (Fitchburg, WI, USA). More details on the generation protocol can be found in [15]. DNA encoding CB_2_R (Uni Prot ID:P34972) flanked with PvuI and XbaI restriction sites (start codon mutated to leucine, internal XbaI site silently mutated) was purchased from Twist BioScience (San Francisco, CA, USA). DNA encoding HER2 (Uni Prot ID:P04626) was from Addgene (plasmid #16257).

DNA was digested with *PvuI* and *XbaI* (New England Biolabs, Ipswich, MA, USA) and ligated into an expression vector containing an IL-6 signal peptide and N-terminal LrgBiT tag (as described in [16]) to create LrgBiT-CB_2_R. For generation of HiBiT-HER2, the HER2 sequence (without its native signal peptide) was amplified using the following primers: forward 5’-GGC GGC TCG AGC GGT GCG ATC ACC CAA GTG TGC ACC GGC-3’; reverse 5’-TGC ATG CCT GCA GGT CGA CTT CAC ACT GGC ACG TCC AG-3’. The N-terminal HiBiT plasmid backbone (generated as described in [16]) was digested with *PvuI* and *XbaI*, and the HER2 PCR product was cloned in using Gibson assembly. To generate LrgBiT-HER2, the HER2 sequence was amplified essentially as described above but with the forward primer 5’-GGC TCG AGC GGT GGG GCG ATC ACC CAA GTG TGC ACC GGC-3’ and cloned in frame with the IL-6 signal peptide into the N-terminal LrgBiT plasmid backbone using Gibson assembly. Plasmids were verified by Oxford Nanopore Sequencing (Plasmidsaurus, London, UK).

HEK293T or CHO-K1 cells were plated into 6-well plates at a density of 5 × 10^5^ (HEK293T) or 2,5 × 10^5^ (CHO-K1) cells/well. The next day cells were transfected at a 1:1 ratio of HiBiT:LrgBiT-tagged receptor plasmids using FuGENE HD at a 3:1 DNA:reagent ratio following manufacturer’s instructions (Promega). The next day, cells were plated at 4 × 10^4^ cells/well into poly-D-lysine-coated (10 μg/ml; Sigma-Aldrich) white, clear, flat bottomed 96-well plates (Grenier Bio-one (655098); Stonehouse, UK). On the fourth day, cells were treated with 10 nM trastuzumab-FITC (Stratech, Ely, UK) prepared in HEPES buffered saline solution (HBSS; 2 mM sodium pyruvate, 145 mM NaCl, 10 mM D-glucose, 5 mM KCl, 1 mM MgSO_4_·7H_2_O, 10 mM HEPES, 1.3 mM CaCl_2_, 1.5 mM NaHCO_3_ in double-distilled water, pH 7.45) supplemented with 0.1% BSA in the presence or absence of trastuzumab (Stratech) for 1 h at 37 °C. Furimazine (Promega) was then added (1:400 final dilution) and luminescence and fluorescence emissions simultaneously measured on the PheraStar FSX (BMG Labtech, Ortenberg, Germany) using an optic module fitted with 475 nm (±30 nm; luminescence detection) and 535 nm (±30 nm; fluorescence detection) band pass filters. BRET ratios were calculated by dividing fluorescence by luminescence emissions.

Binding affinities (K_i_) of trastuzumab were calculated using the Cheng-Prusoff equation:

$${K}_{i}=\frac{{{IC}}_{50}}{1+\,\frac{[L]}{{K}_{D}}}$$

where [L] was the concentration of trastuzumab-FITC used (nM), IC_50_ the molar concentration of trastuzumab that inhibited 50% of the specific binding of trastuzumab-FITC, and K_D_ (nM) the affinity of trastuzumab-FITC determined in saturation binding experiments (HiBiT-HER2—LrgBiT-HER2 K_D_ = 5.73 nM; HiBiT-HER2—LrgBiT-CB_2_R K_D_ = 13.17 nM; HiBiT-EGFR—LrgBiT-HER2 K_D_ = 5.29 nM; data not shown). Data were normalized for each experimental replicate to total binding of 10 nM trastuzumab-FITC (100%) and vehicle (0%).

### Enzyme-linked immunosorbent assay (ELISA)

IFN-γ levels in the cancer cell + NK cell co-cultures were assessed by using the Human IFN-γ Standard TMB ELISA Development Kit (PeproTech, Cranbury, NJ, USA) following manufacturer’s instructions. Briefly, co-cultures were established as described in the Cell viability assays section, and culture media collected 24 h later. Conditioned media was incubated for 2 h in 96-well, flat-bottom ELISA microplates previously coated with anti-human IFN-γ antibody. Detection was carried out by subsequent incubation with a detection antibody and streptavidin-HRP conjugate. Color development was monitored with an ELISA plate reader.

### Animals

All animal procedures were conducted in accordance with European regulations, with the approval of the Complutense University Animal Experimentation Committee and the Madrid Regional Government (PROEX 150.2/23). The maximal tumor burden permitted was 1 × 10^3^ mm^3^ and it was never exceeded. Animals were housed in the UCM School of Biology animal facility under a 12-h light-dark cycle, with ad libitum access to food and water. Sample size was calculated using the GRANMO Sample Size Calculator (version 8), assuming a two-sided test with an alpha risk of 0.05 and a beta risk of 0.2, a common standard deviation of 1.4, and an estimated 20% dropout rate.

Xenografts were generated by subcutaneous injection of 2 × 10⁶ TR or TR CB_2_R cells (resuspended in Matrigel) into the right flank of 6-week-old female athymic nude-Foxn1nu mice (RRID:IMSR_ENV:HSP-069, Envigo, Barcelona, Spain). Due to estrogen receptor (ER) expression in BT474 cells, drinking water was supplemented with 17β-estradiol (Sigma-Aldrich) starting one week before cancer cell inoculation and continuing until sacrifice. Tumors were monitored regularly using a digital caliper, and their volume was calculated as (4π/3) × (width/2)² × (length/2). Once tumors reached a volume of 100–200 mm³, animals were randomly assigned to experimental groups by sequential allocation to the different treatment arms without additional selection criteria, and treatment commenced. Blinding was not implemented during treatment administration, as knowledge of group allocation was required to ensure correct dosing and treatment delivery.

For **xenografts derived from TR cells**, six treatment groups were established with the following schedules: Trastuzumab (20 mg/kg in saline, intraperitoneally (IP), twice weekly); Erlotinib (50 mg/kg in PEG/saline 1:1, IP, five times weekly); Erlotinib followed by trastuzumab (One week of erlotinib followed by three weeks of trastuzumab); Trastuzumab followed by erlotinib (One week of trastuzumab followed by three weeks of erlotinib); Trastuzumab + erlotinib (Four weeks of combined treatment); Vehicle (PEG/saline, IP, five times weekly).

For **xenografts derived from TR CB**_**2**_**R cells**, two groups were established: Trastuzumab and Vehicle, following the same dosing schedules as above.

After four weeks of treatment (or earlier if tumor volumes exceeded 1×10^3 ^mm³), mice were euthanized. Tumors were excised and divided into two portions when possible—one for protein extraction and the other for RNA isolation—and snap-frozen until subsequent processing.

### Statistical analyses

Sample size was determined based on prior experience using similar HER2+ breast cancer models. No samples or animals were excluded from the analyses.

Pearson’s chi-squared test was used for statistical analysis of the human samples included in the TMAs. Kaplan-Meier survival curves were statistically compared by the log-rank test. For the rest of data, normality was assessed using the Shapiro-Wilk test. For normally distributed datasets, independent two-group comparisons were performed using Student’s *t* test, while for multi-group analyses one-way or two-way ANOVA followed by Tukey’s post hoc test were used. Non-normally distributed data were analyzed via the Mann–Whitney *U* test for two independent groups or the Kruskal-Wallis test with Dunn’s post hoc correction for multi-group comparisons. All statistical tests were two-tailed, with significance thresholds set at *p* < 0.05. Unless otherwise stated, data are expressed as mean ± SEM. Statistical analysis was performed with GraphPad Prism version 8.0.1. or 10.4.2.

## Results

### A reduction in HER2-CB_2_R expression after NAT is linked to treatment resistance

**Fig. 1:**
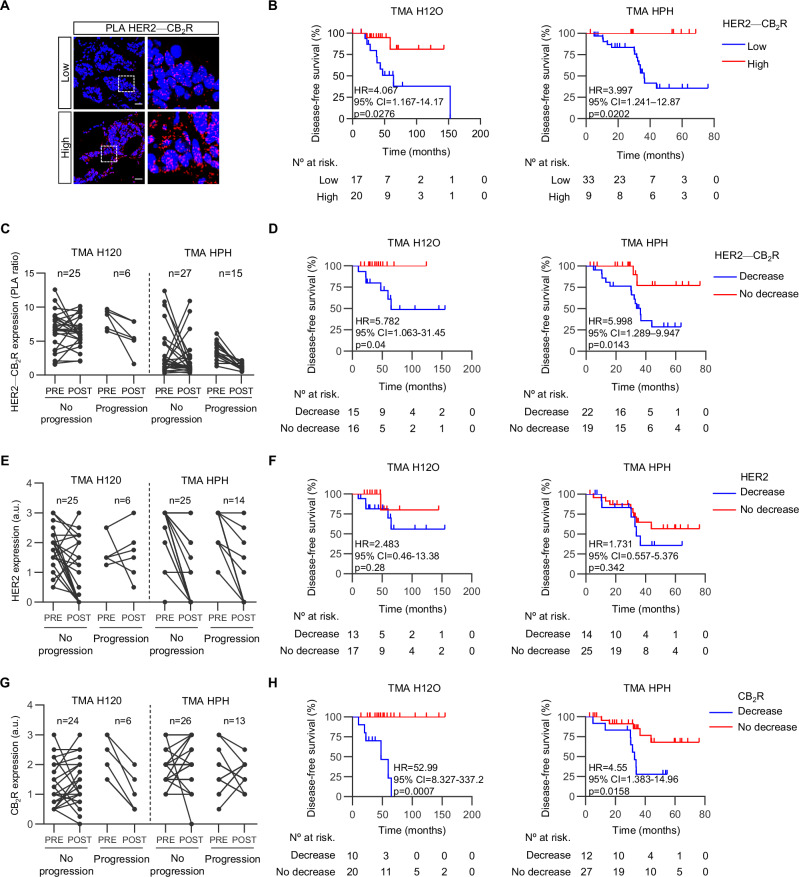
A reduction in HER2-CB_2_R and CB_2_R expression after NAT is linked to treatment resistance. **A** Representative Proximity Ligation Assay (PLA) images of human samples exhibiting high and low HER2-CB_2_R heteromer expression. Red: PLA signal; blue: nuclear staining (DAPI). Scale bar, 25 μm. **B** Kaplan-Meier plots of disease-free survival for samples obtained after NAT and included in the tissue microarrays from Hospitals 12 de Octubre (TMA H12O) and Puerta de Hierro (TMA HPH). **C** Relative HER2-CB_2_R expression, as determined by PLA, in matched TMA samples. The left data columns in each panel represent samples from patients with no long-term disease progression, while the right columns represent the non-responders. Lines connect paired samples from the same patient before (PRE) and after (POST) NAT. **D** Kaplan-Meier plots of disease-free survival, stratified by type of change in HER2-CB_2_R dimer expression after NAT, in samples from the indicated TMAs. HER2 (**E**) and CB_2_R (**G**) expression, as determined by IHC, in samples from TMA H12O and TMA HPH, represented as indicated in panel (**C**). Kaplan-Meier plots of disease-free survival, stratified by changes in HER2 (**F**) or CB_2_R expression (**H**) after NAT, in samples from the indicated TMAs.

To investigate the potential involvement of the HER2-CB_2_R heterodimer in trastuzumab resistance, we analyzed its expression by proximity ligation assays (PLAs) in two tissue microarrays (TMAs) containing two independent cohorts of human HER2+ tumor samples obtained after NAT, which included trastuzumab in all cases. Patients with lower disease-free survival, indicating a poorer response to therapy and/or an increased resistance, had lower HER2-CB_2_R levels compared to responsive patients, which had higher long-term survival (Fig. 1A, B). Our previous work had found that high HER2-CB_2_R expression at initial biopsy (before NAT) is associated with poor patient prognosis and, therefore, more aggressive phenotypes [11]. Together, these findings led us to hypothesize that treatment resistance might be linked to a decrease in HER2-CB_2_R expression after NAT. Analysis of 73 matched pre- and post-NAT samples supported this idea: patients who responded to therapy (i.e., with no long-term disease progression) showed varied changes in HER2-CB_2_R expression (upregulation, downregulation, or no change), while those with treatment failure (i.e., with disease progression) consistently showed a decline in HER2-CB_2_R expression after NAT (Fig. 1C). This drop was further associated with lower disease-free survival (Fig. 1D).

The separate analysis of HER2 and CB_2_R expression in these samples (Supp. Figure. S1) indicated that the decrease in HER2-CB_2_R after NAT was primarily due to reduced CB_2_R levels. Thus, we did not observe a consistent change in HER2 levels in patients with or without disease progression (Fig. 1E). Consequently, changes in HER2 expression after NAT were not associated with differences in disease-free survival (Fig. 1F). Regarding CB_2_R expression, changes after NAT mirrored those of the heteromer in one of the TMAs (H12O): no clear pattern in responders (i.e., those without long-term disease progression) and a decrease in non-responders (i.e., those with disease progression) (Fig. 1G). However, in the other sample collection (HPH), this association was not observed (Fig. 1G). Nonetheless, a reduction in CB_2_R levels after NAT was associated with lower disease-free survival in both patient cohorts (Fig. 1H). These findings collectively suggest that CB_2_R and HER2-CB_2_R may serve as novel predictors of long-term response to treatment, and that the decrease in HER2-CB_2_R dimers following NAT may contribute to the development of treatment resistance. Nonetheless, the prognostic value of the proposed biomarkers should be interpreted with caution owing to the relatively small sample size and the limited number of events in each cohort, which constrained the complexity of multivariable survival analyses. These limitations warrant confirmation in larger, independent cohorts.

### CB_2_R loss leads to trastuzumab resistance

**Fig. 2:**
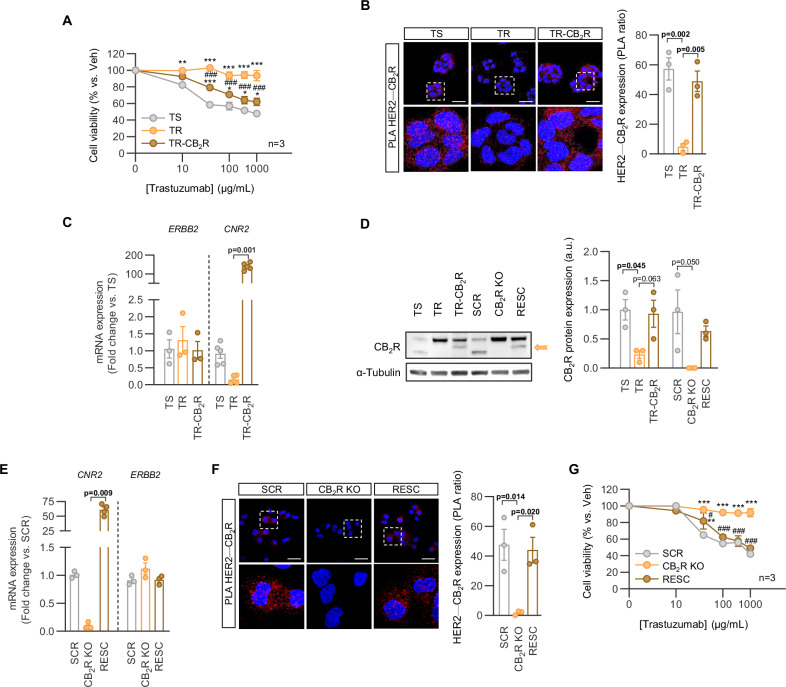
Loss of CB_2_R leads to trastuzumab resistance. **A** Viability of BT474-derived cell lines in response to trastuzumab. TS: trastuzumab-sensitive; TR: trastuzumab-resistant; TR-CB_2_R: TR with overexpression of CB_2_R. **B** Representative PLA images of HER2-CB_2_R (left) and quantification of PLA signal (right, n = 3). Red: PLA signal; blue: nuclear staining (DAPI). Scale bar, 50 μm. **C**, **E** mRNA expression of HER2 (*ERBB2*) and CB_2_R (*CNR2*) in the indicated cell lines, as determined by qPCR. SCR: Scramble (TS cells); CB_2_R KO: TS cells with genetic deletion of CB_2_R; RESC: CB_2_R KO cells reinfected with CB_2_R. **D** CB_2_R expression, as determined by Western blot. The densitometric analysis is shown next to representative luminograms. **F** Representative HER2-CB_2_R PLA images (left) and quantification (right). Scale bar, 50 μm. **G** Cell viability in response to trastuzumab treatment for 5 days. All data, except for those in panels (**C**) and (**E**) (*CNR2*), were normally distributed and analyzed using two-tailed ANOVAs [two-way in panels (**A**) and (**G**); one-way in panels (**B**), (**D**), (**E**) (*ERBB2*), and (**F**)]. Non-normally distributed data were analyzed using the Kruskal-Wallis test. * *p* < 0.05, ** *p* < 0.01, *** *p* < 0.01 vs. TS (A) or SCR (G); # *p* < 0.05, ### *p* < 0.001 vs. TR (**A**) or CB_2_R KO (**G**).

We examined the hypothesis that a reduced HER2-CB_2_R heterodimer formation post-NAT drives treatment resistance using a pair of BT474-derived breast cancer cell lines, one sensitive (TS) and another resistant (TR) to trastuzumab (Fig. 2A). Consistent with our previous observations (Fig. 1), resistant cells exhibited lower levels of HER2-CB_2_R (Fig. 2B) and CB_2_R (Fig. 2C, D). We also found a cause-effect link between reduced HER2-CB_2_R expression due to decreased CB_2_R and trastuzumab resistance: re-expression of CB_2_R in resistant cells restored HER2-CB_2_R levels and trastuzumab sensitivity (Fig. 2A–D), and genetic silencing of CB_2_R in sensitive cells decreased HER2-CB_2_R levels and induced trastuzumab resistance, effects that were both reversed upon CB_2_R re-expression (Fig. 2D–G). Trastuzumab resistance linked to reduced HER2-CB_2_R and CB_2_R expression was further validated in a HER2+ breast cancer patient-derived xenograft (PDX) model, PDX118, consisting of a TS and TR pair of cell lines (Supplementary Fig. S2A). Thus, TR cells showed decreased HER2-CB_2_R (Fig. Supplementary S2B) and CB_2_R expression (Fig. Supplementary S2C and D) when compared to their corresponding TS counterparts.

**Fig. 3:**
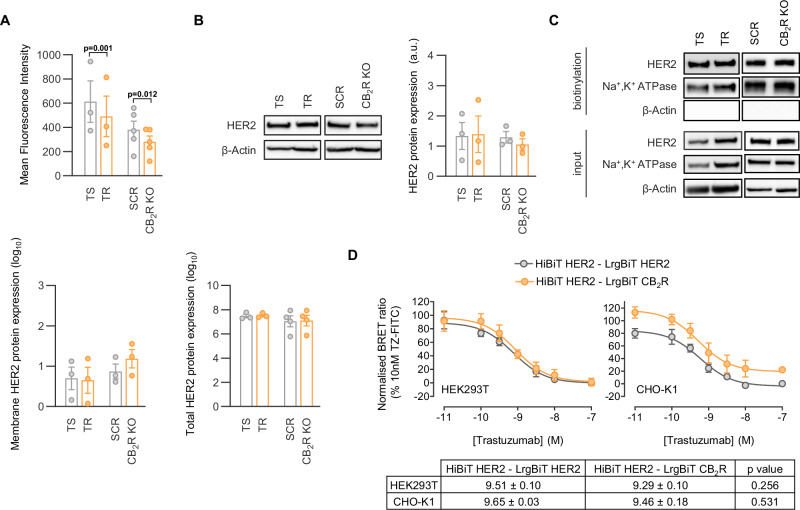
Trastuzumab resistance does not depend on differential affinity for HER2-HER2 versus HER2-CB_2_R dimers. **A** Binding of trastuzumab conjugated to fluorescein isothiocyanate (trastuzumab-FITC) to HER2, as determined by flow cytometry, in the indicated cell lines. Representative luminograms of protein expression analysis of total HER2 by Western blot (**B**) and of membrane-bound HER2 by biotinylating (**C**). Densitometry values were normalized to β-Actin (**B**), or to both Na⁺/K⁺-ATPase and β-Actin (**C**) and are presented in the graphs on the right (**B**) or below (**C**). **D** Competition NanoBiT ligand-binding studies between trastuzumab-FITC (10 nM) and unlabeled trastuzumab to HiBiT-HER2—LrgBiT-HER2 (gray circles) or HiBiT-HER2—LrgBiT-CB_2_R (orange circles) expressed in HEK293T (left panel; *n* = 5) or CHO-K1 (right panel; *n* = 3) cells. Data are expressed as % of total trastuzumab-FITC binding in the absence of competitor (100%) and vehicle alone (0%). pKi values are shown in the table below the graphs. All data were normally distributed and were analyzed by Student’s *t* test or one-way ANOVA (**D**).

To elucidate trastuzumab resistance mechanisms associated with CB_2_R loss, we first examined whether a reduced HER2 availability -potentially caused by decreased HER2-CB_2_R complex formation– may underlie therapeutic resistance. In line with this idea, trastuzumab-resistant cells (TR and CB_2_R KO) exhibited significantly reduced trastuzumab-FITC binding than their sensitive counterparts (TS and SCR, respectively) (Fig. 3A). This was not due to a decrease in HER2 expression, as mRNA (Fig. 2C, E), total protein (Fig. 3B), and plasma membrane-located protein (Fig. 3C) were all comparable across cell lines. The reduced trastuzumab binding to resistant cells was not due to a decreased affinity either. Thus, ligand displacement experiments in HEK293T and CHO-K1 cells expressing NanoBiT-tagged HER2 and CB_2_R revealed nearly identical pKi values for trastuzumab in cells transfected with HER2-HER2 or HER2-CB_2_R complementary vectors (Fig. 3D), demonstrating comparable binding affinity regardless of CB_2_R presence. Hence, these findings point to the existence of alterations in trastuzumab-to-HER2 binding associated with trastuzumab resistance, and to the relevance of CB_2_R expression as a key mediator in this process.

### Trastuzumab resistance linked to reduced CB_2_R is due to evasion of antitumor IFN-γ signaling

**Fig. 4:**
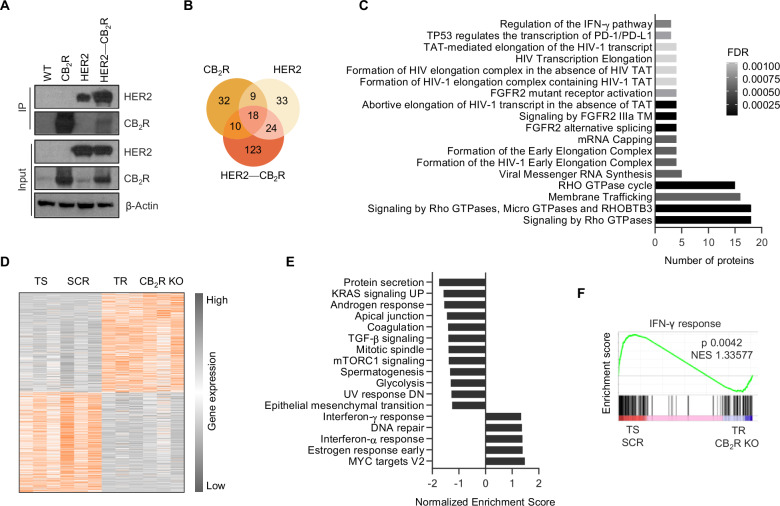
Identification of signaling routes involved in trastuzumab resistance due to reduced HER2-CB_2_R. **A** Representative co-immunoprecipitation (co-IP) blot of proteins from HEK293T wild-type (WT) cells and WT-derived lines stably expressing HER2, HA-tagged CB_2_R or both. **B** Venn diagram illustrating proteins identified as potential interactors of CB_2_R, HER2, or the HER2-CB_2_R complex using PD software. **C** Key signaling pathways modulated by the 123 proteins selectively interacting with the HER2-CB_2_R dimer, as identified by Reactome software. **D** Relative expression of differentially expressed genes in trastuzumab-sensitive (TS and SCR) and resistant (TR and CB_2_R KO) cell lines, determined by RNA sequencing (RNAseq). **E** Oncogenesis-related gene signatures differentially expressed between sensitive and resistant cell lines. Results are shown as variation against the resistant lines and therefore positive values indicate pathways overrepresented in sensitive lines, while negative values indicate pathways underrepresented in sensitive lines (and thus overrepresented in resistant cells). **F** Gene Set Enrichment Analysis (GSEA) of the RNAseq samples. The green line represents the enrichment ratio. NES normalized enrichment score.

To investigate whether trastuzumab resistance involves the activation of compensatory signaling pathways in our models, we first conducted a proteomic study in HEK293T cells expressing HA-CB_2_R and HER2, either individually or together (Fig. 4A). We identified a set of 32 proteins potentially interacting with CB_2_R, a set of 33 proteins potentially interacting with HER2, and a set of 123 potentially interacting proteins when both receptors were co-expressed (Fig. 4B and Supplementary Table S1). This suggests the existence of a unique HER2-CB_2_R interactome and, therefore, of a potential heteromer-driven signaling distinct from monomeric signaling. Using Reactome software, we found that these 123 proteins modulate key cancer-related pathways, including interferon-γ (IFN-γ), fibroblast growth factor receptor (FGFR), and small G protein signaling (Fig. 4C).

**Fig. 5:**
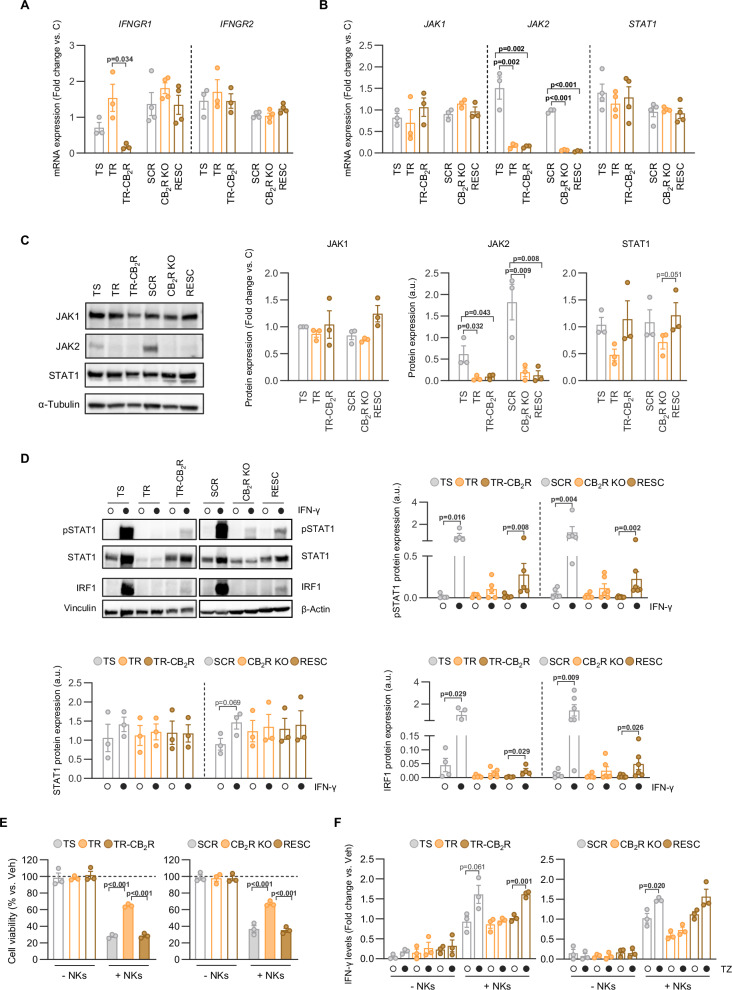
CB_2_R loss-driven trastuzumab resistance is associated with impaired antitumor IFN-γ signaling. mRNA (**A**, **B**) and protein (**C**, **D**) expression, as determined by qPCR and Western blot, respectively, of different components of the IFN-γ/JAK/STAT pathway in the indicated cell lines. The densitometric analysis of the WB experiments in (**C**, **D**) is shown next to representative luminograms. In (**D**), cells were exposed to IFN-γ for 6 h. **E** Cancer cell viability following 24 h co-culture with the NK cell line NK92-CD16-GFP and 1 h trastuzumab exposure. Control groups (-NK cells; first three bars in each panel) represent cancer cells cultured in the absence of NK cells. The viability of the corresponding vehicle-treated cells was set at 100% and is represented as a horizontal dotted line. **F** IFN-γ levels in the extracellular medium of the co-cultures described in (**E**), as determined by ELISA. All data, except for those in panels (**A**) (*IFNGR1* TS group), (**C**) (STAT1) and **D** (pSTAT1 and IRF1) were normally distributed and analyzed using two-tailed one-way ANOVA and a Student’s *t* test [in (**F**)]. Non-normally distributed data were analyzed using the Kruskal-Wallis test.

To explore the relevance of a HER2-CB_2_R-driven signaling in trastuzumab resistance, we performed RNA sequencing (RNAseq) on our cancer cell models. This analysis revealed differential expression of 8983 genes between trastuzumab-sensitive and trastuzumab-resistant cells (Fig. 4D). Notably, 17 genetic signatures related to oncogenesis differed significantly between the two groups (Fig. 4E). The IFN-γ response signature was one of them and appeared underrepresented in resistant cell lines. Gene set enrichment analysis (GSEA) confirmed this finding, showing reduced expression of this signature in trastuzumab-resistant cells (Fig. 4F). These observations suggest that trastuzumab resistance may involve impaired responsiveness to IFN-γ antitumor signaling. However, a comprehensive pathway analysis revealed no consistent alterations in the key elements of this route that were CB_2_R-dependent. For example, no significant differences were observed between sensitive and resistant cell lines in the mRNA expression of IFN-γ receptors (Fig. 5A and Supplementary Fig. S2E). Regarding the downstream JAK/STAT proteins, qPCR (Fig. 5B and Supplementary Fig. S2F) and Western blot (Fig. 5C and Supplementary Fig. S2G) experiments showed similar JAK1 expression in all cell lines. JAK2 mRNA and protein levels appeared reduced in both BT474-derived trastuzumab-resistant cell lines (TR and CB_2_R KO) compared to their sensitive counterparts (TS and SCR, respectively) (Fig. 5B, C), as well as in the PDX-derived TR model (Supplementary Fig. S2F and 2G), but CB_2_R re-expression did not bring its expression back to basal levels (Fig. 5B, C). Regarding STATs, we focused on STAT1, a crucial mediator of IFN-γ signaling, and the data did not show any significant difference in its mRNA (Fig. 5B and Supplementary Fig. S2F) or protein levels (Fig. 5C and Supplementary Fig. S2G) between cell lines.

Although we were not able to identify any basal differences in the expression of key IFN-γ signaling components between cell lines that could account for CB_2_R-dependent trastuzumab resistance, we found a distinct IFN-γ response profile between cell groups. Thus, upon IFN-γ stimulation, sensitive cells were able to activate this cascade, increasing the active form of STAT1 (pSTAT1) and the downstream target IRF1. In contrast, resistant cells showed minimal changes in these proteins (Fig. 5D). Notably, the sensitivity to IFN-γ was dependent on CB_2_R, as its re-expression in resistant cells restored their capability to activate the pathway, thus enhancing STAT1 and IRF1 expression (Fig. 5D).

NK cells are a primary source of IFN-γ in tumors and key effectors in ADCC, a pivotal mechanism of trastuzumab’s antitumor action. Specifically, NK cells recognize trastuzumab-coated tumor cells through the interaction of trastuzumab with CD16 (FcγRIIIA) receptors on their surface, thus triggering the release of cytotoxic molecules from lytic granules, as well as proinflammatory cytokines, including IFN-γ, ultimately inducing target cell death [17]. Co-cultures of our cancer cells with a CD16 + NK cell line showed signs of impaired ADCC in resistant cells. In these experiments, and to isolate ADCC effects, we restricted trastuzumab exposure to 1 h and assessed viability at 24 h—conditions under which trastuzumab monotherapy alone failed to induce cytotoxicity in cancer cell monocultures, regardless of sensitivity status (Fig. 5E). In this setting, sensitive cells exhibited marked trastuzumab-induced cytotoxicity, suggesting functional ADCC activation. In contrast, resistant cells maintained high viability in the co-cultures, indicative of a defective ADCC response (Fig. 5E). These effects were accompanied by an impaired release of IFN-γ to the extracellular medium in co-cultures with resistant cells (Fig. 5F). The pivotal role of CB_2_R in these differential responses was evidenced by the impaired ADCC-associated features that were observed in cells lacking CB_2_R and were restored upon CB_2_R overexpression (Fig. 5E, F).

Collectively, these findings indicate that trastuzumab resistance associated with reduced CB_2_R is driven, at least partially, by impaired NK-cell mediated cytotoxicity and evasion of antitumor IFN-γ signaling.

### Trastuzumab resistance due to reduced CB_2_R expression relies on the formation of alternative HER2 receptor heterodimers

**Fig. 6:**
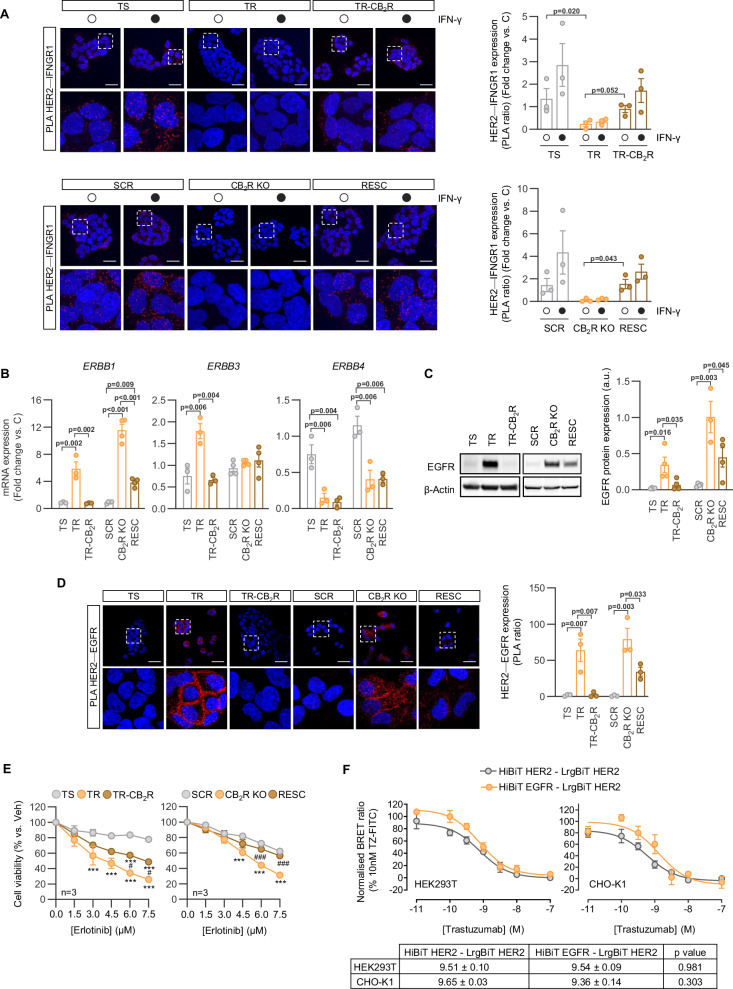
Trastuzumab resistance driven by CB_2_R downregulation relies on HER2 heterodimer reorganization. **A** Representative HER2-IFNGR1 PLA images (left) and quantification (right) in the BT474-derived cells before and after IFN-γ treatment. Red: PLA signal; blue: nuclear staining (DAPI). Scale bar, 50 μm. **B** mRNA expression levels, determined by qPCR, of HER receptor family members. **C** Representative Western blot analysis (left) and densitometry quantification (right) of EGFR expression. **D** Representative HER2-EGFR PLA images (left) and quantification (right) in the BT474-derived cell lines. **E** Viability of the indicated cell lines in response to the selective EGFR inhibitor erlotinib. **F** Competition NanoBiT ligand-binding studies between trastuzumab-FITC (10 nM) and unlabeled trastuzumab to HiBiT-HER2 – LrgBiT-HER2 (gray circles) or HiBiT-EGFR—LrgBiT-HER2 (orange circles) expressed in HEK293T (left panel; *n* = 5) or CHO-K1 (right panel; *n* = 3) cells. Data are expressed as % of total trastuzumab-FITC binding in the absence of competitor (100%) and vehicle alone (0%). All data were normally distributed and analyzed using two-tailed ANOVAs [two-way in panel (E); one-way in the rest of panels]. *** *p* < 0.01 vs. TS (left) or SCR (right); # *p* < 0.05, ### *p* < 0.001 vs. TR (left) or CB_2_R KO (right).

A well-established mechanism of trastuzumab resistance involves the formation of alternative receptor heterodimers. By engaging in dimerization with other members of the HER family or with other RTKs, cancer cells reduce their reliance on the original HER2-containing dimers for growth and survival signaling, thereby evading trastuzumab’s inhibitory effects [9]. We observed that resistance to trastuzumab linked to evasion of antitumor IFN-γ signaling was associated with a decrease in HER2-IFNGR1 heterodimers (Fig. 6A and Supplementary Fig. S2H), an effect that was dependent on CB_2_R expression (Fig. 6A). Furthermore, IFN-γ stimulation increased the formation of HER2-IFNGR1 heterodimers in sensitive but not in resistant cells (Fig. 6A). These observations support that CB_2_R loss-mediated trastuzumab resistance stems from impaired responsiveness to IFN-γ´s antitumor signaling.

Our results also revealed a shift in the expression profile of HER family receptors between TS and TR cells. This altered pattern in TR cells was characterized by changes in individual HER members, including increased EGFR expression in BT474-based models (Fig. 6B, C) and elevated HER3 levels in the PDX118-derived model (Supplementary Fig. S2I, J). This observation was accompanied by an increased HER2-EGFR or HER2-HER3 heterodimer formation (Fig. 6D and Supplementary Fig. S2K). As predicted, the dimer switch to HER2-EGFR made trastuzumab-resistant cells more dependent on EGFR pro-survival signaling. Thus, resistant cells showed heightened sensitivity to the antiproliferative effect of erlotinib (a selective EGFR inhibitor) (Fig. 6E) and lapatinib (a dual HER2/EGFR inhibitor) (Supplementary Fig. S3A), and this signaling shift was CB_2_R dependent, as the increase in EGFR levels (Fig. 6B, C), HER2-EGFR dimerization (Fig. 6D) and erlotinib sensitivity (Fig. 6E) occurred in response to CB_2_R loss and were prevented upon CB_2_R re-expression. In line with these observations, tucatinib, a HER2-selective inhibitor that does not target EGFR, produced comparable effects in trastuzumab-sensitive and -resistant cells (Supplementary Fig. S3B). These results further support the notion that trastuzumab resistance is associated with a shift toward EGFR dependency and suggest that dual HER2/EGFR inhibition may represent an effective therapeutic strategy in CB_2_R-low, trastuzumab-resistant contexts.

We also investigated whether trastuzumab resistance could result from reduced affinity of trastuzumab for HER2 when dimerized with EGFR, as opposed to HER2-HER2 homodimers. Displacement assays in HEK293T and CHO-K1 cells overexpressing either HER2-HER2 or HER2-EGFR complexes demonstrated that this was not the case, as trastuzumab exhibited virtually identical affinity for both dimeric forms (Fig. 6F).

**Fig. 7:**
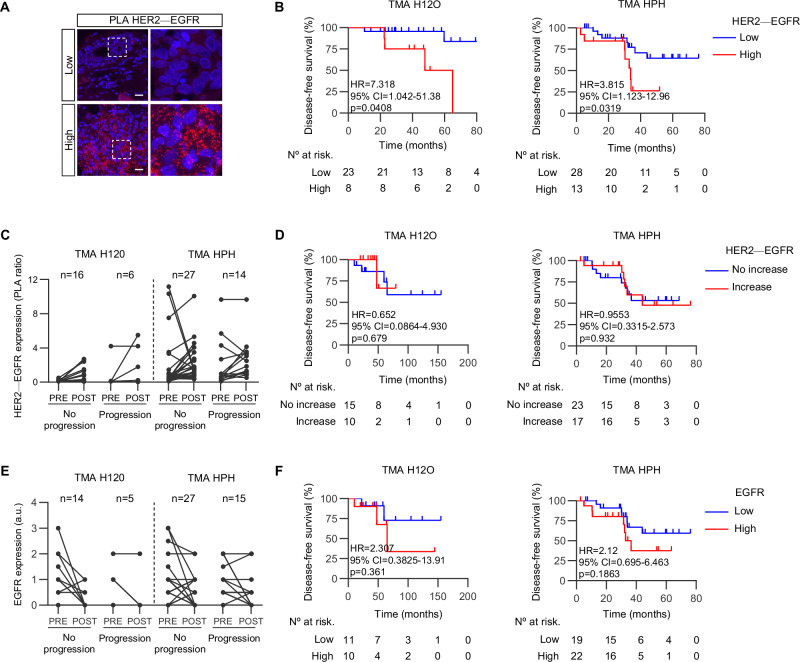
High post-NAT HER2-EGFR heterodimer burden predicts poor treatment outcomes. **A** Representative Proximity Ligation Assay (PLA) images of human samples exhibiting high and low HER2-EGFR heteromer expression. Red: PLA signal; blue: nuclear staining (DAPI). Scale bar, 50 μm. **B** Kaplan-Meier plots of disease-free survival for samples obtained after NAT and included in the tissue microarrays from Hospitals 12 de Octubre (TMA H12O) and Puerta de Hierro (TMA HPH). **C** Relative HER2-EGFR expression, as determined by PLA, in matched TMA samples. The left data columns in each panel represent samples from patients with no long-term disease progression, while the right columns represent the non-responders. Lines connect paired samples from the same patient before (PRE) and after (POST) NAT. **D** Kaplan-Meier plots of disease-free survival, stratified by type of change in HER2-EGFR dimer expression after NAT, in samples from the indicated TMAs. **E** Relative EGFR expression, as determined by PLA, in matched TMA samples before (PRE) and after NAT (POST). Data are represented as in (**C**). **F** Kaplan-Meier plots of disease-free survival, stratified by type of change in EGFR expression after NAT.

In line with our observations pointing to a signaling switch towards HER2-EGFR signaling in our trastuzumab resistance contexts, elevated HER2-EGFR heterodimer expression in human samples after NAT was significantly associated with reduced overall patient survival (Fig. 7A, B), indicating increased treatment resistance. As opposed to HER2-CB_2_R, the changes in HER2-EGFR expression (Supplementary Fig. S1) before and after NAT did not follow any pattern with predictive value (Fig. 7C, D), and EGFR expression alone after NAT did not show predictive significance either (Fig. 7E, F). Collectively, these findings further support the relevance of a receptor dimer shift as a pivotal mechanism underlying the acquisition of trastuzumab resistance, and suggest that monitoring HER2-EGFR dimer expression after NAT could provide valuable prognostic insights into therapeutic resistance and patient outcomes.

### Trastuzumab resistance linked to reduced CB_2_R is overcome by targeting EGFR and HER2 signaling and by non-CB_2_R-selective cannabinoid treatment

**Fig. 8:**
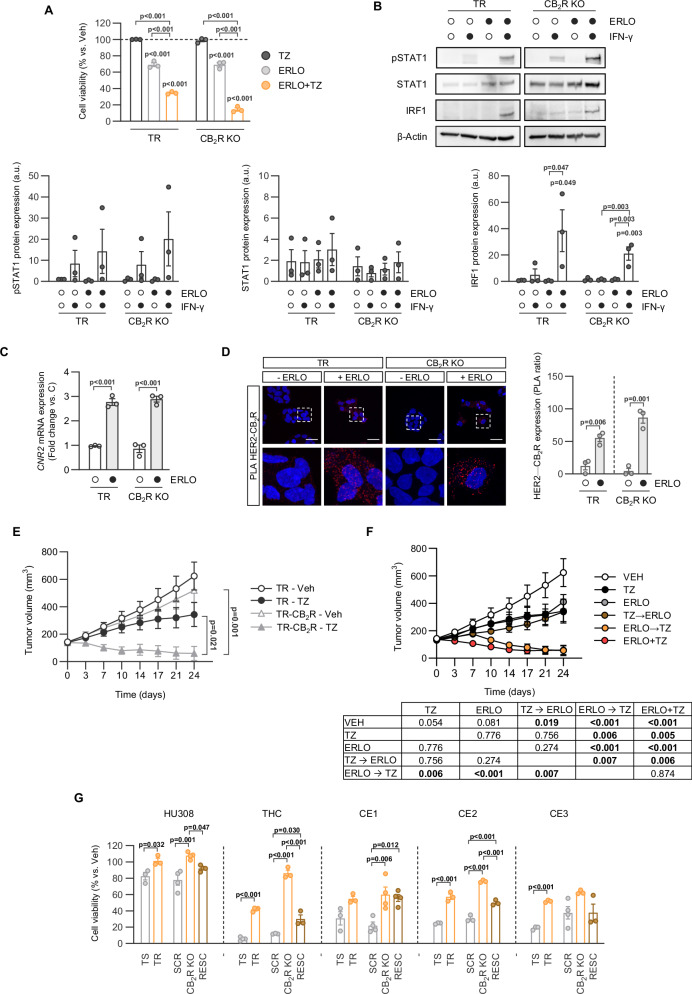
Trastuzumab resistance linked to reduced CB_2_R is overcome by targeting EGFR and HER2 signaling, and by non-CB_2_R-selective cannabinoid treatments. **A** Viability of the indicated cell lines in response to trastuzumab and erlotinib treatment (alone or in combination) for 4 days. The viability of the corresponding vehicle-treated cells was set at 100% and is represented as a dotted horizontal line. **B** Representative Western blot (top) and densitometric quantification (bottom) of protein expression profiles following a 3-day treatment with erlotinib (5 μM), IFN-γ (20 ng/mL, 6 h) or the combination. **C** CB_2_R mRNA expression, as determined by qPCR in response to erlotinib treatment. **D** Representative HER2-CB_2_R PLA images (left) and quantification (right). Red: PLA signal; blue: nuclear staining (DAPI). Scale bar, 50 μm. **E**, **F** Tumor growth in nude female mice bearing subcutaneous xenografts generated by injection of the indicated cell lines. **G** Viability of the indicated cell lines in response to the CB_2_R-selective agonist HU308, THC (a CB_1_R/CB_2_R-mixed agonist) and three different cannabis preparations (CE1, CE2 and CE3) for 1 day. The viability of the corresponding vehicle-treated cells was set at 100%. CE1 and CE2 were THC rich extracts, while CE3 contained a 1:1 THC:CBD ratio. The precise composition of the three CEs is shown in Supplementary Table S2. All data were normally distributed and analyzed using two-tailed one-way ANOVAs (**A**, **B**, and **G**) or Student’s *t* test (**C**–**G**). In (**A**) and (**B**), the *p* values right above the bars correspond to comparisons with vehicle-treated cells.

The identification of a signaling switch responsible for trastuzumab resistance led us to hypothesize that targeting the newly emerged pro-oncogenic driver (EGFR) could overcome this resistance. This notion was subsequently validated both in vitro and in vivo. First, the combination of trastuzumab and erlotinib significantly reduced the viability of trastuzumab-resistant cells in culture, with this reduction exceeding that produced by erlotinib alone (Fig. 8A). These findings suggest a synergistic effect that may result from trastuzumab resensitation. This mechanism was further substantiated by the two following observations: first, erlotinib treatment restored the functionality of the JAK/STAT signaling pathway in trastuzumab-resistant cells, as evidenced by IFN-γ recovering its ability to modulate pSTAT1 and IRF1 levels (Fig. 8B). Second, erlotinib administration in resistant cells reestablished both CB_2_R expression (Fig. 8C) and HER2-CB_2_R heterodimer formation (Fig. 8D).

In vivo studies provided further support to the critical role of CB_2_R in trastuzumab resistance and validated that targeting EGFR signaling represents a promising therapeutic strategy to overcome this resistance. Thus, CB_2_R overexpression in trastuzumab-resistant cells successfully restored sensitivity to trastuzumab antitumor action. Strikingly, in many animals, we observed not only an impaired tumor growth but a complete tumor regression (Fig. 8E). Furthermore, simultaneous targeting of EGFR (with erlotinib) and HER2 (with trastuzumab) effectively reversed treatment resistance, resulting in numerous complete tumor regressions (Fig. 8F). Similar efficacy was achieved when erlotinib treatment preceded trastuzumab administration, but not when the sequence was reversed (i.e., trastuzumab followed by erlotinib) (Fig. 8F), further supporting the idea that EGFR inhibition restores trastuzumab sensitivity.

Cannabinoids are well known to induce antitumor responses in preclinical cancer models [18, 19]. In HER2+ breast cancer, these effects are largely mediated by activation of CB_2_R [20]. We therefore hypothesized that trastuzumab-resistant cells with reduced/absent CB_2_R expression would be less/no responsive to therapies based on CB_2_R-selective activation. Accordingly, the CB_2_R-selective agonist HU308 reduced cell viability in trastuzumab-sensitive models (TS, SCR and RESC), whereas this effect was attenuated/abolished in resistant cells with low/absent CB_2_R expression (TR and CB_2_R KO) (Fig. 8G). These findings suggest that trastuzumab-resistant tumors with low CB_2_R levels are unlikely to respond to antitumor strategies based solely on CB_2_R activation. However, this does not exclude cannabinoids as potential therapeutic agents, as certain cannabinoid compounds and *Cannabis sativa* preparations exert antitumor effects through additional molecular targets. To explore this possibility, resistant cells were treated with Δ^9^-tetrahydrocannabinol (THC), a CB_1_R/CB_2_R-mixed agonist, and three distinct *Cannabis sativa* extracts, including two THC-rich preparations and one with a 1:1 THC:cannabidiol (CBD) ratio. In addition to the indicated cannabinoids, these extracts contained other minor cannabinoids and terpenes (Supplementary Table S2). In contrast to HU308, these treatments -although to a lesser extent- also reduced viability in trastuzumab-resistant cells (Fig. 8G). Together, these results suggest that cannabinoids may retain antitumor activity in trastuzumab-resistant HER2⁺ breast cancer through CB_2_R-independent mechanisms.

Collectively, these data suggest that HER2 + BC patients who exhibit decreased HER2-CB_2_R heterodimers and CB_2_R expression after NAT, accompanied by increased EGFR levels and HER2-EGFR dimerization, have a higher likelihood of developing resistance to anti-HER2 treatment in the long term. These patients could potentially benefit from therapeutic strategies that simultaneously target both HER2 and EGFR signaling pathways and by cannabinoids that do not selectively target CB_2_R.

## Discussion

HER2 + BC is one of the tumor types that has significantly benefited from the development of targeted therapies. However, important clinical challenges remain unaddressed. While early-stage HER2+ disease responds reasonably well to anti-HER2 treatments, metastatic disease continues to be associated with disease progression in most cases. This therapeutic limitation is primarily attributable to resistance mechanisms, both innate (de novo) and adaptive (acquired), that ultimately compromise treatment effectiveness.

In the context of cancer, both cannabinoid receptors and their endogenous ligands are typically overexpressed compared to healthy tissue. However, contradictory data suggest that alterations in this system are context- and tumor type-dependent [21]. Specifically, an association between cannabinoid receptor expression and tumor progression has been established in BC. Thus, decreased CB_1_R mRNA levels and increased CB_2_R mRNA levels were found in tumor tissue compared to non-tumor mammary epithelial tissue, and the expression of CB_2_R was associated with tumor aggressiveness (histological grade) [22]. A subsequent analysis in a larger sample collection revealed that the vast majority of HER2+ breast tumors express CB_2_R [20]. This pointed to a functional connection between CB_2_R and HER2, as it was further demonstrated. Thus, HER2 induces the expression of CB_2_R, which then forms heterodimers with HER2, stabilizing it at the plasma membrane by preventing its proteasomal degradation and favoring HER2-driven oncogenic signaling [12]. Directly related to this observation, it has also been reported that the HER2-CB_2_R heterodimer has prognostic value in HER2 + BC (its expression in tumor samples obtained before any treatment correlates with poor patient prognosis), and that its disruption triggers antitumor effects [11].

Here, we demonstrate that HER2-CB_2_R heterodimers are also involved in modulating responsiveness to trastuzumab. Specifically, we show that loss of CB_2_R induces a shift in HER2-containing heterodimers, resulting in cancer cells that are less sensitive to the antitumor effects of trastuzumab. Recent studies have established that receptor heterodimerization is a widespread and pivotal mechanism of modulating cell signaling. These dimers have been described not only among RTKs (whose mechanism of activation involves dimerization), but also between GPCRs and even between RTKs and GPCRs [23–25]. The formation of these complexes enhances cellular versatility in response to stimuli, as the signaling properties of receptor heterodimers frequently differ from those of the individual receptors. For CB_2_R, heteromers with other GPCRs relevant in cancer, such as CXCR4 [26] or GPR55 [27], have been reported. In these cases, complex formation facilitates cross-antagonism between the protomers.

In this study, we observed that trastuzumab resistance is triggered by a shift in the cancer cell receptor heterodimer pattern. Specifically, the loss of CB_2_R leads to a decrease in HER2-CB_2_R heterodimers that is accompanied by a reduction in HER2-IFNGR1 and an increase in HER2-EGFR expression. This signaling switch makes cancer cells less dependent on HER2 and less responsive to IFN-γ antitumor signaling, while increasing their reliance on EGFR-driven growth and survival pathways. This type of signaling switch is a well-described mechanism by which cancer cells evade the pressure of anticancer drugs and is a common strategy for the acquisition of trastuzumab resistance. Thus, the overexpression of other HER family members (mainly EGFR and HER3), as well as other non-HER RTKs (IGF-1R, c-MET, EphA2, AXL), and non-RTK receptors (EpoR, ER, β2-AR), has been associated to trastuzumab resistance [9, 28]. For example, IGF-1R has been implicated in trastuzumab resistance by forming heterodimers with HER2, which leads to the phosphorylation and activation of HER2 upon IGF-1 binding. Of interest, targeting IGF-1R disrupts the HER2-IGF-1R interaction and restores sensitivity to trastuzumab [29]. Our findings reveal an increase in HER2-EGFR heterodimers with a shift toward EGFR-dependent signaling. The link between trastuzumab resistance and EGFR overexpression is well established in both preclinical models and human BC cases of HER2 + BC. Moreover, this overexpression has been associated with modulation of HER2/EGFR homo- and heterodimerization dynamics, and the dual inhibition of EGFR and HER2 has proved more effective than monospecific agents in overcoming trastuzumab resistance [28]. In fact, several clinical trials have evaluated the antitumor efficacy of dual HER2/EGFR targeting by combining trastuzumab with EGFR inhibitors (such as erlotinib and gefitinib), EGFR-HER2 dual inhibitors (such as lapatinib), pan-HER inhibitors (neratinib, pyrotinib), or anti-EGFR monoclonal antibodies (cetuximab). However, routine clinical practice does not systematically analyze EGFR expression to guide treatment decisions. Our study supports the importance of that systematic analysis as the trastuzumab resistance mechanism described herein may be overcome by simultaneous targeting of HER2 and EGFR.

In addition to an increase in HER2-EGFR dimerization, we report that trastuzumab resistance due to CB_2_R loss produces a decrease in HER2-IFNGR1 heterodimers. The functional interplay between HER2 and the IFN-γ signaling pathway is well established. For example, IFN-γ reduced plasma membrane HER2 levels (which is associated with diminished growth and induction of tumor senescence) by promoting its proteasomal degradation. Moreover, the combination of anti-HER2 antibodies with IFN-γ synergistically decreased surface HER2 expression and impaired tumor growth in an animal model of BC [30]. Furthermore, disruption of IFN-γ signaling by genetic ablation of IFNGR1 conferred resistance to HER2-targeted cell bispecific antibodies and CAR-T cells [31]. Of interest, here we report the existence of HER2-IFNGR1 heterodimers and their involvement in trastuzumab resistance. Specifically, we found that BC cells that become resistant to trastuzumab due to a decrease in CB_2_R express less HER2-IFNGR1 dimers and are less responsive to IFN-γ. Although the detailed mechanistic basis for this effect remains to be elucidated, our findings suggest that the physical interaction between HER2 and IFNGR1 modulates the signaling properties of both receptors.

Our data also suggest that trastuzumab resistance due to CB_2_R loss is mediated, at least in part, by blocking the ability of NK cells to induce ADCC, a pivotal mechanism underlying the antitumor activity of trastuzumab. NK cells recognize trastuzumab-opsonized tumor cells via their activating CD16 (FcγRIIIa) receptors, triggering their cytotoxic machinery and leading to the release of cytotoxic molecules such as IFN-γ, ultimately resulting in target cell death [4]. A link between trastuzumab resistance and evasion of NK-cell mediated ADCC has been previously described, and several strategies are being explored to boost this immune response and, consequently, overcome trastuzumab resistance [4, 17]. One of these strategies involves optimizing trastuzumab structure by genetically engineering its Fc domain to increase binding to FcγR. However, the monoclonal antibody margetuximab, developed with this approach, did not show therapeutic advantage over trastuzumab in overall survival of patients with previously treated HER2+ advanced BC in the SOPHIA study [32]. Other experimental approaches involve boosting NK cell activity and infiltration, or blocking ADCC inhibitory signals, but none have been routinely implemented in clinical practice yet [4, 17]. In this case, combination therapies are also being considered as a potential means to overcome ADCC evasion and the resulting trastuzumab resistance. Various chemotherapeutic drugs (including anthracyclines, cyclophosphamide, and taxanes) that are used together with or in sequence after HER2-targeting antibody therapies induce immunogenic cell death (ICD), a specific form of apoptosis that directly counteracts ADCC evasion mechanisms. ICD is characterized by the release of damage-associated molecular patterns (DAMPs) such as HMGB1, which has been shown to enhance NK cell activation and recruitment to tumors. In addition, in vitro treatment with anthracyclines and taxanes enhanced anti-HER2 monoclonal antibody-induced ADCC by promoting endoplasmic reticulum stress and upregulating NKG2D-ligands in BC cells [4, 17].

Our findings also highlight combination approaches as a promising strategy to overcome trastuzumab resistance due to CB_2_R loss. On the one hand, we have demonstrated that simultaneous (or sequential) targeting of EGFR and HER2 resensitizes resistant cells to the antitumor effect of trastuzumab. In addition, previous studies have shown that cannabinoids elicit antitumor responses in preclinical models of cancer, including HER2 + BC, suggesting the potential benefit of combining them with trastuzumab. The antitumor effects of cannabinoids rely on their ability to inhibit cancer cell proliferation, induce their apoptotic death, suppress angiogenesis, and reduce the invasive and migratory capacities of tumor cells, thereby lowering their metastatic potential [18, 19]. The vast preclinical research consistently demonstrating these effects has set the bases for the clinical study of these compounds as antitumor agents in glioblastoma.

In summary, this study identifies a novel mechanism underlying trastuzumab resistance, in which the ECS —specifically through CB_2_R— plays a pivotal role. In addition, our findings highlight the functional significance of heteromeric receptor patterns in cancer cells, suggesting that, analogous to molecular signatures associated with BC subtypes, heterodimer signatures may possess predictive value. Finally, elucidation of this mechanism supports the potential development of new therapeutic strategies to overcome resistance based on the simultaneous or sequential targeting of EGFR and HER2, reinforcing the potential clinical utility of routine EGFR expression analysis to optimize treatment selection for individual patients.

## Supplementary information

Supplementary Material

## Data Availability

The data generated in this study are available within the article and its additional data files. The proteomic datasets generated in HEK293T cells are available in PRoteomics IDEntifications Database (PRIDE, accession number PXD065700), and the RNAseq datasets generated BT474-derived cell lines in Gene Expression Omnibus (GEO, accession number GSE300749).
