# Supplementary material for: Cannabinoid CB2 receptor drives trastuzumab resistance and predicts durable anti-HER2 response
Source: Oncogene. 2026 May 11;45(25):2436–51. doi: 10.1038/s41388-026-03814-9 (PMC13269139; doi:10.1038/s41388-026-03814-9)
Supplement: Supplementary file 1 — Supplementary Material [file 41388_2026_3814_MOESM1_ESM.pdf]

Supplementary Fig. S1. Representative immunohistochemistry images.

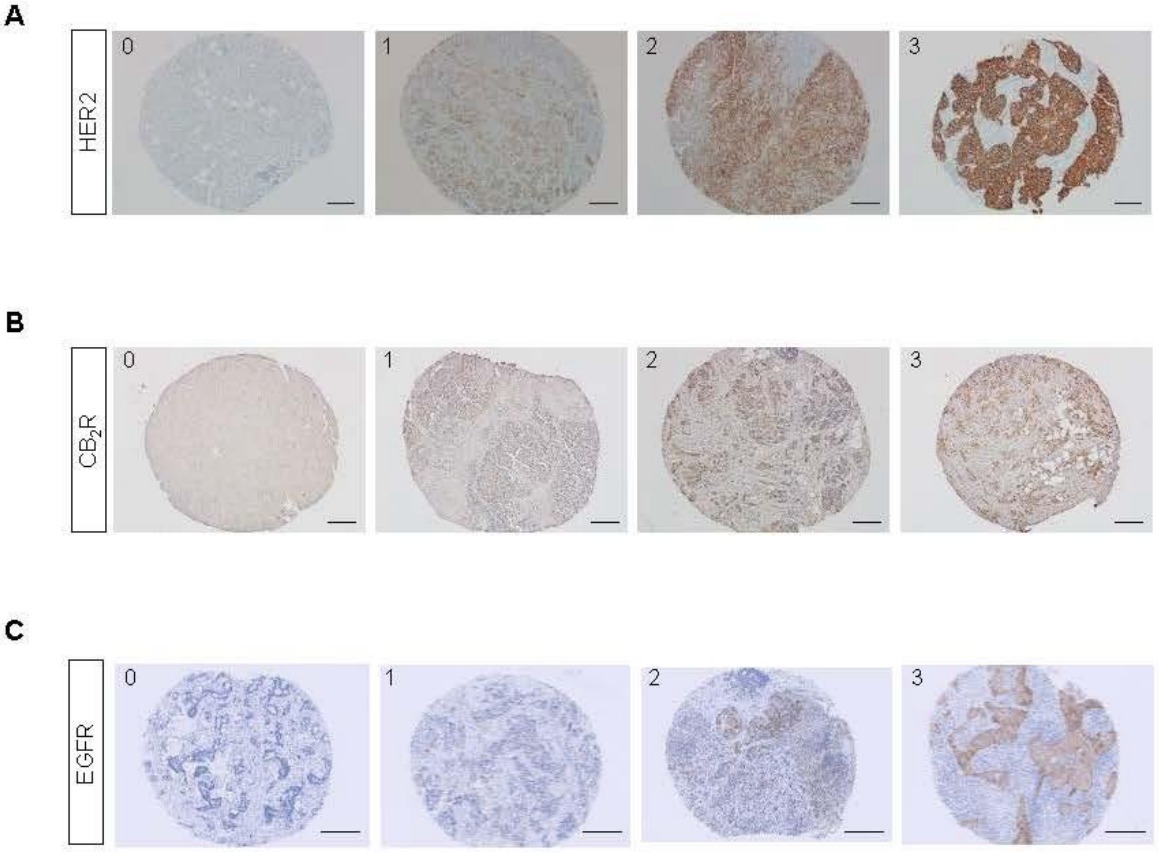

Supplementary Fig. S1. Representative immunohistochemistry images. Representative immunohistochemistry (IHC) images of HER2 (A), CB_2R (B) and EGFR expression (C) in samples from the TMAs from Hospitals 12 de Octubre and Puerta de Hierro, with staining intensities indicated in arbitrary units. Scale bar, 100 μm (A) and (B) and 250 μm (C).

Supplementary Fig. S2. Validation of key findings in an additional HER2+ model.

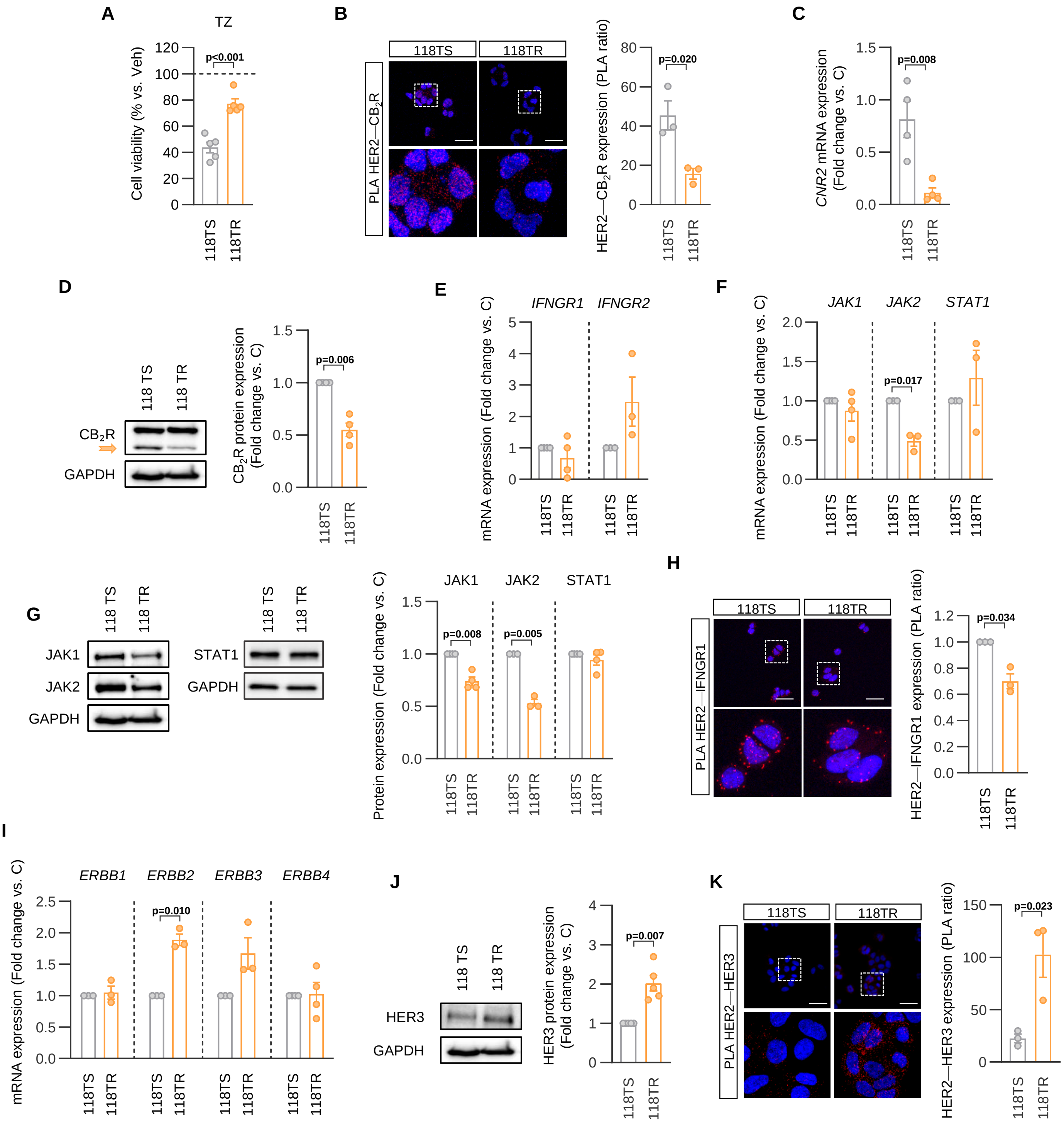

Supplementary Figure S2. Validation of key findings in an additional HER2+ model. (A) Viability of PDX118-derived trastuzumab-sensitive (118TS) and -resistant (118TR) cell lines following treatment with trastuzumab (20 µg/mL). Viability is expressed relative to vehicle-treated controls, set to 100%. (B) Representative proximity ligation assay (PLA) images showing HER2–CB_2R heterodimers (left) and corresponding quantification of PLA signal (right; n = 3). (C–G) Expression of the indicated genes and proteins, as assessed by qPCR (C, E, F) and Western blot analysis (D, G). (H) Representative PLA images of HER2–IFNGR1 complexes (left) and quantification of PLA signal (right; n = 3). (I–J) Expression of the indicated genes and proteins determined by qPCR (I) and Western blot (J). (K) Representative PLA images of HER2–HER3 dimers

(left) and quantification of PLA signal (right; n = 3). For all PLA images, red indicates PLA signal and blue indicates nuclear staining (DAPI). Scale bars, 50 μm. All data were normally distributed and analyzed using two-tailed two-way ANOVA (A); Student's *t*-test (B, C, and K); or one-sample *t*-test (D-J).

Supplementary Fig. S3. Effect of small-size tyrosine kinase inhibitors on trastuzumab-sensitive and -resistant cells.

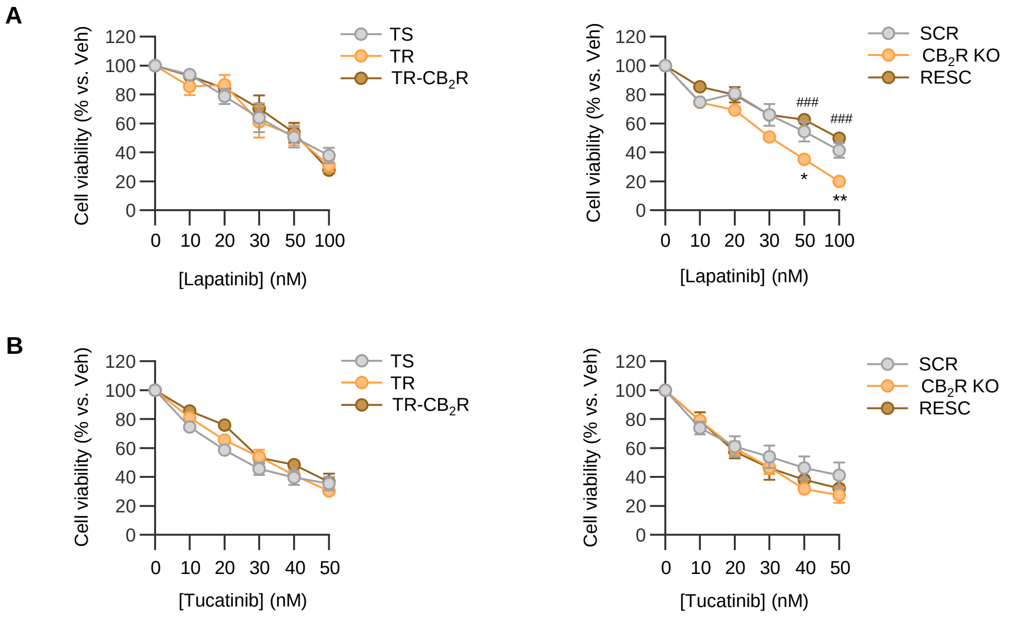

Supplementary Figure S3. Effect of small-size tyrosine kinase inhibitors on trastuzumab-sensitive and -resistant cells. Viability of the indicated cell lines in response to the dual HER2/EGFR inhibitor lapatinib (A) and the HER2-selective inhibitor tucatinib (B). Viability is expressed relative to vehicle-treated controls, set to 100%. All data were normally distributed and analyzed using two-tailed two-way ANOVA (A-B). * $p < 0.05$, ** $p < 0.01$ vs. SCR (A); ### $p < 0.001$ vs. CB_2R KO (A).

Supplementary Table S1. Potential proteins interacting with the HER2-CB2R heterodimer.

List of proteins identified as potential interactors of CB2R, HER2 or the HER2-CB2R dimer, as determined by proteomic analysis in HEK293T cells. The main proposed function was obtained from the GeneCards Database. The full proteomic dataset is available in PRoteomics IDEntifications Database (PRIDE, accession number PXD065700).

	Accession	Abbreviation	Description	Main function
CB$_2$R-interacting proteins	P19823	ITIH2	Inter-alpha-trypsin inhibitor heavy chain H2	Serine protease inhibitor involved in extracellular matrix stabilization and suppression of tumor metastasis.
	Q99720	OPRS1	Sigma 1-type opioid receptor	Receptor interacting with psychotomimetic drugs such as cocaine and amphetamines, implicated in endocrine, immune, and nervous system functions.
	Q8WUY1	CH055	UPF0670 protein C8orf55	UPF0670 family protein; function unknown. Highly expressed in cancers.
	P30536	TSPO	Translocator protein	Key regulator of cholesterol transport into mitochondria, initiating steroid hormone biosynthesis.
	A0A0B4J1U7	HV601	Immunoglobulin heavy variable 6-1	Predicted antigen-binding protein involved in immunoglobulin-mediated immune responses.
	Q9BQ75	CC026	Uncharacterized protein C3orf26	Poorly characterized protein of unknown function.
	O15127	SCAMP2	Secretory carrier-associated membrane protein 2	SCAMP family member involved in post-Golgi recycling and vesicular transport to the plasma membrane.
	Q8NFP7	NUDT10	Diphosphoinositol polyphosphate phosphohydrolase 3-alpha	Phosphohydrolase regulating diphosphoinositol polyphosphate turnover and intracellular trafficking.
	P04114	APOB	Apolipoprotein B-100	Major apolipoprotein of chylomicrons and LDL; ligand for the LDL receptor involved in lipid transport.
	P13535	MYH8	Myosin-8	Class II myosin heavy chain functioning in skeletal muscle contraction.
	O14545	TRAFD1	TRAF-type zinc finger domain-containing protein 1	Negative feedback regulator of innate immune signaling, preventing excessive inflammatory responses.
	Q9Y623	MYH4	Myosin-4	Double-stranded RNA-binding protein involved in muscle contraction.
	O95861	BPNT1	3'(2'),5'-bisphosphate nucleotidase 1	Magnesium-dependent bisphosphate 3′-nucleotidase involved in nucleotide metabolism and inositol signaling.
	Q9Y4R8	TELO2	Telomere length regulation protein TEL2 homolog	S-phase checkpoint protein involved in cell cycle control and DNA repair.
	Q7L1Q6	BZW1	Basic leucine zipper and W2 domain-containing protein 1	RNA- and cadherin-binding protein regulating translational initiation.
	P06732	CKM	Creatine kinase M-type	Energy homeostasis enzyme and serum marker of myocardial infarction. Catalyzes phosphagen phosphate transfer.
	Q9UNH7	SNX6	Sorting nexin-6	PX-domain–containing nexin family protein involved in intracellular trafficking and receptor signaling.
	Q9UI30	TR112	tRNA methyltransferase 112 homolog	Protein methyltransferase and adaptor involved in macromolecule methylation and metabolic regulation.
	Q9BRA2	TXNDC17	Thioredoxin domain-containing protein 17	Redox enzyme with peroxidase and disulfide reductase activity involved in TNF-mediated signaling.
	Q92538	GBF1	Golgi-specific brefeldin A-resistance guanine nucleotide exchange factor 1	Golgi-localized guanine nucleotide exchange factor activating ARF1 to regulate vesicular trafficking.
	O43819	SCO2	Protein SCO2 homolog, mitochondrial	Cytochrome c oxidase assembly factor involved in electron transfer and mitochondrial respiration.
	P43307	SSRA	Translocon-associated protein subunit alpha	Translocon-associated protein alpha (TRAPα); facilitates ER protein translocation and folding.
	P10768	ESTD	S-formylglutathione hydrolase	S-formylglutathione hydrolase. Hydrolyzes S-formylglutathione to formate and glutathione.
	Q5VYK3	ECM29	Proteasome-associated protein ECM29 homolog	Regulator of proteasome activity and protein degradation.
	P61011	SRP54	Signal recognition particle 54 kDa protein	7S RNA–binding protein involved in ER targeting, GTPase activity, and granulocyte differentiation.
	P15927	RPA2	Replication protein A 32 kDa subunit	Subunit of the replication protein A complex essential for DNA replication, repair, recombination, and ATR-mediated DNA damage response.
	O00151	PDLIM1	PDZ and LIM domain protein 1	Adaptor protein linking LIM-interacting proteins to the cytoskeleton; involved in muscle development.
	Q14331	FRG1	Protein FRG1	RNA-binding protein with actin-bundling activity involved in mRNA processing and muscle cytoskeletal organization.
	P26583	HMGB2	High mobility group protein B2	DNA-bending protein facilitating cooperative protein–DNA interactions and chromatin organization.
	O14929	HAT1	Histone acetyltransferase type B catalytic subunit	Histone acetyltransferase involved in acetylation of newly synthesized histones during chromatin assembly.
	Q15942	ZYX	Zyxin	Signal transduction mediator regulating adhesion-dependent gene expression and actin cytoskeleton organization.
	Q01130	SRSF2	Splicing factor, arginine/serine-rich 2	SR-rich spliceosome component essential for pre-mRNA splicing, mRNA export, and translation.
HER2-interacting proteins	Q15274	NADC	Nicotinate-nucleotide pyrophosphorylase [carboxylating]	Sodium-coupled citrate transporter regulated by cyclophilin B.
	Q96F44	TRIM11	E3 ubiquitin-protein ligase TRIM11	E3 ubiquitin ligase mediating protein ubiquitination and proteasomal degradation.
	Q8IY85	CQ057	EF-hand domain-containing protein C17orf57	Predicted calcium-binding protein potentially involved in signaling or structural regulation.
	O95563	BR44	Brain protein 44	Cell adhesion molecule regulating synaptic plasticity and neuronal migration.
	P56556	NDUFA6	NADH dehydrogenase [ubiquinone] 1 alpha subcomplex subunit 6	Accessory subunit of mitochondrial complex I involved in electron transport.
	Q15392	DHCR24	24-dehydrocholesterol reductase	FAD-dependent oxidoreductase required for cholesterol biosynthesis.
	Q15041	AR6P1	ADP-ribosylation factor-like protein 6-interacting protein 1	Transmembrane protein involved in protein transport and membrane trafficking during myeloid hematopoietic maturation.
	Q9BTE7	DCNL5	DCN1-like protein 5	Cullin scaffold protein acting in the neddylation pathway to regulate ubiquitin ligase activity.
	Q9UN37	VPS4A	Vacuolar protein sorting-associated protein 4A	ATPase involved in endosomal sorting and multivesicular body formation.
	O94808	GFPT2	Glucosamine--fructose-6-phosphate aminotransferase [isomerizing] 2	Glutamine–fructose-6-phosphate transaminase involved in UDP-N-acetylglucosamine biosynthesis.
	P46926	GNPDA1	Glucosamine-6-phosphate isomerase 1	Allosteric enzyme catalyzing reversible glucosamine-6-phosphate deamination.
	P27361	MAPK3	Mitogen-activated protein kinase 3	MAP kinase regulating proliferation, differentiation, and cell cycle progression.
	Q16822	PCK2	Phosphoenolpyruvate carboxykinase [GTP], mitochondrial	Catalyzes oxaloacetate conversion to phosphoenolpyruvate in gluconeogenesis.
	Q96G23	LASS2	LAG1 longevity assurance homolog 2	Ceramide synthase involved in sphingolipid metabolism, cell growth, and apoptosis.
	O95837	GNA14	Guanine nucleotide-binding protein subunit alpha-14	Gαq family protein activating phospholipase C-β signaling pathways.
	Q9H845	ACAD9	Acyl-CoA dehydrogenase family member 9, mitochondrial	Rate-limiting enzyme of mitochondrial fatty acid β-oxidation.
	O60547	GMDS	GDP-mannose 4,6 dehydratase	Catalyzes the first step in GDP-fucose biosynthesis from GDP-mannose.
	Q13363	CTBP1	C-terminal-binding protein 1	Transcriptional repressor involved in cell proliferation and Polycomb-mediated gene regulation.
	Q9NRX2	MRPL17	39S ribosomal protein L17, mitochondrial	Structural protein of the mitochondrial large ribosomal subunit.
	O75436	VPS26A	Vacuolar protein sorting-associated protein 26A	Retromer complex component mediating endosome-to–trans-Golgi retrograde transport.
	Q96A35	MRPL24	39S ribosomal protein L24, mitochondrial	Component of the mitochondrial 39S ribosomal subunit involved in translation.
	P10398	ARAF	Serine/threonine-protein kinase A-Raf	Serine/threonine kinase regulating apoptosis, TOR signaling, and protein metabolism.
	O75489	NDUFS3	NADH dehydrogenase [ubiquinone] iron-sulfur protein 3, mitochondrial	Core subunit of mitochondrial complex I mediating electron transfer from NADH to ubiquinone.
	Q14318	FKBP8	Peptidyl-prolyl cis-trans isomerase FKBP8	Immunophilin family protein involved in immunoregulation and fundamental cellular processes, including protein folding and trafficking.
	Q9BTE3	CJ119	UPF0557 protein C10orf119	Poorly characterized; predicted to be involved in cellular stress response.
	Q9Y3C6	PPIL1	Peptidyl-prolyl cis-trans isomerase-like 1	Immunophilin required for protein folding, cyclosporin A–mediated immunosuppression, and HIV-1 infection.
	Q00059	TFAM	Transcription factor A, mitochondrial	Mitochondrial transcription factor essential for mtDNA replication and repair.
	A6NIZ1	RAP1BL	Ras-related protein Rap-1b-like protein	Rap-family small GTPase regulating cAMP signaling and synaptic vesicle exocytosis.
	Q9UG63	ABCF2	ATP-binding cassette sub-family F member 2	ABC transporter involved in transmembrane transport and cancer progression.
	Q9UHV9	PFDN2	Prefoldin subunit 2	Prefoldin complex subunit assisting folding of newly synthesized polypeptides.
	Q8WW12	PCNP	PEST proteolytic signal-containing nuclear protein	Protein involved in proteasome-mediated ubiquitin-dependent protein catabolic process and protein ubiquitination.
	Q9UHD8	SEPT9	Septin-9	Protein involved in cytokinesis and cell cycle regulation.
	P55036	PSMD4	26S proteasome non-ATPase regulatory subunit 4	Ubiquitin receptor directing ubiquitinated proteins to the proteasome.
	O60499	STX10	Syntaxin-10	Syntaxin-family SNARE protein involved in vesicle docking and fusion at the Golgi apparatus.
	P15260	IFNGR1	Interferon gamma receptor 1	Interferon-γ receptor that activates JAK/STAT signaling in immune responses.
	P27037	ACVR2A	Activin receptor type-2A	Mediator of activin signaling, members of the TGF-β superfamily involved in diverse biological processes.
	P36896	ACVR1B	Activin receptor type-1B	Receptor serine/threonine kinase for TGF-β/activin family ligands.
	P43250	GRK6	G protein-coupled receptor kinase 6	G protein–coupled receptor kinase that phosphorylates activated GPCRs, promoting receptor desensitization.
	P55082	MFAP3	Microfibril-associated glycoprotein 3	Extracellular matrix microfibril component with structural functions.
	Q5VT40	FAM78B	Protein FAM78B	Putative regulatory or accessory protein with unknown specific function.
	Q7Z4F1	LRP10	Low-density lipoprotein receptor-related protein 10	Involved in internalization of lipophilic molecules and/or signal transduction.
	Q86VP1	TAX1BP1	Tax1-binding protein 1	Interacts with TNFAIP3 to inhibit TNF-induced apoptosis and inflammatory signaling.
	Q9NY26	SLC39A1	Zinc transporter ZIP1	Zinc transporter mediating cytosolic zinc uptake; associated with cancer and neurodegenerative disease.
	Q9Y397	ZDHHC9	Palmitoyltransferase ZDHHC9	Palmitoyltransferase that specifically palmitoylates HRAS and NRAS.
	Q9Y5R8	TRAPPC1	Trafficking protein particle complex subunit 1	Component of the TRAPP complex involved in ER-to-Golgi vesicular transport.
	A1KXE4	FAM168B	Protein FAM168B	Predicted regulator of axonogenesis and gene expression.
	O14763	TNFRSF10B	Tumor necrosis factor receptor superfamily member 10B	Death receptor activated by TRAIL (TNFSF10) that transduces apoptotic signals.
	O95249	GOSR1	Golgi SNAP receptor complex member 1	Golgi SNARE complex component mediating protein trafficking between ER and Golgi compartments.
	Q13137	CALCOCO2	Calcium-binding and coiled-coil domain-containing protein 2	Autophagy receptor for ubiquitin-coated bacteria, essential for innate immune responses.
	Q16539	MAPK14	Mitogen-activated protein kinase 14	MAP kinase involved in signal integration controlling proliferation, differentiation, transcription, and development.
	Q6P1M0	SLC27A4	Long-chain fatty acid transport protein 4	Mediates cellular uptake of long-chain fatty acids.
	Q86Y82	STX12	Syntaxin-12	SNARE-related protein involved in autophagosome assembly, cholesterol efflux, and protein stabilization.
	Q8N357	C2orf18	Transmembrane protein C2orf18	Poorly characterized, likely membrane-associated protein with regulatory function.
	Q8WUM9	SLC20A1	Sodium-dependent phosphate transporter 1	Sodium–phosphate symporter involved in phosphate uptake for cellular metabolism and biosynthesis.

HER2-CB_2R-interacting proteins

Q96DZ1	ERLEC1	Endoplasmic reticulum lectin 1	ER-associated degradation regulator promoting cellular stress responses and metastatic survival.
Q96ER3	SAAL1	Serum Amyloid A Like 1	Acts upstream of positive regulation of synoviocyte proliferation.
Q96IJ6	GMPPA	Mannose-1-phosphate guanyltransferase alpha	GDP-mannose pyrophosphorylase involved in N-linked oligosaccharide biosynthesis.
Q99735	MGST2	Microsomal glutathione S-transferase 2	Catalyzes leukotriene C4 synthesis, a key mediator of inflammation.
Q9NRP0	OSTC	Oligosaccharyltransferase complex subunit OSTC	Adaptor protein involved in co-translational protein modification and N-linked glycosylation; component of OST complex A.
Q8IYS1	PM20D2	Peptidase M20 domain-containing protein 2	Dipeptidase involved in proteolysis and regulation of protein metabolism.
Q9H223	EHD4	EH domain-containing protein 4	Cadherin-binding protein involved in endocytic recycling and protein homooligomerization.
O00186	STXBP3	Syntaxin-binding protein 3	Syntaxin-binding protein that negatively regulates calcium-dependent exocytosis, neutrophil degranulation, and platelet aggregation.
O43464	HTRA2	Serine protease HTRA2, mitochondrial	Mitochondrial quality control protease with pro-apoptotic functions.
O95864	FADS2	Fatty acid desaturase 2	Fatty acid desaturase regulating unsaturation of fatty acyl chains.
P49137	MAPKAPK2	MAP kinase-activated protein kinase 2	Stress-activated kinase regulated by p38 MAPK, involved in inflammation, gene expression, nuclear export, and proliferation.
P62330	ARF6	ADP-ribosylation factor 6	Small GTPase regulating vesicular trafficking, membrane lipid remodeling, and actin cytoskeleton dynamics.
Q08752	PPID	Peptidyl-prolyl cis-trans isomerase D	Peptidyl-prolyl cis–trans isomerase accelerating protein folding.
Q53FV1	ORMDL2	ORM1-like protein 2	Involved in ceramide metabolism and negative regulation of ceramide biosynthesis.
Q9Y241	HIGD1A	HIG1 domain family member 1A	Acts upstream of negative regulation of apoptotic processes.
O75886	STAM2	Signal transducing adapter molecule 2	Modulates signaling downstream of JAK kinases following cytokine stimulation.
P09669	COX6C	Cytochrome c oxidase subunit 6C	Terminal enzyme of the mitochondrial respiratory chain catalyzing electron transfer from cytochrome c to oxygen.
P53350	PLK1	Serine/threonine-protein kinase PLK1	Key mitotic kinase regulating spindle formation, mitosis, and cytokinesis.
Q15386	UBE3C	Ubiquitin-protein ligase E3C	E3 ubiquitin ligase involved in K29- and K48-linked ubiquitination and ubiquitin-dependent protein catabolic processes.
Q6NUK1	SLC25A24	Calcium-binding mitochondrial carrier protein SCaMC-1	Mitochondrial inner membrane transporter of small molecules, including ornithine.
Q99986	VRK1	Serine/threonine-protein kinase VRK1	Nuclear kinase that phosphorylates p53 and regulates cell cycle progression, chromatin organization, DNA repair, and proliferation.
O75251	NDUFS7	NADH dehydrogenase [ubiquinone] iron-sulfur protein 7, mitochondrial	Participates in electron transfer from NADH to ubiquinone in the mitochondrial respiratory chain.
P62070	RRAS2	Ras-related protein R-Ras2	Small Ras-family GTPase involved in signal transduction, cell proliferation, and differentiation.
P62875	POLR2L	DNA-directed RNA polymerases I, II, and III subunit RPABC5	Core subunit shared by RNA polymerases I, II, and III, essential for transcription.
Q9NRG9	AAAS	Aladin	Nuclear pore complex protein involved in nucleocytoplasmic transport and nuclear envelope integrity.
O75352	MPDU1	Mannose-P-dolichol utilization defect 1 protein	Involved in dolichol-linked oligosaccharide assembly required for N-glycosylation.
O95168	NDUFB4	NADH dehydrogenase [ubiquinone] 1 beta subcomplex subunit 4	Subunit of mitochondrial NADH dehydrogenase (complex I), contributing to complex structure and stability.
P51809	VAMP7	Vesicle-associated membrane protein 7	SNARE protein mediating vesicle fusion, exocytosis, and endosomal trafficking.
Q7Z3U7	MON2	Protein MON2 homolog	Predicted role in membrane trafficking and Golgi–endosome dynamics.
Q96L46	CAPNS2	Calpain small subunit 2	Calcium-dependent cysteine-type endopeptidase.
Q9BPW8	NIPSNAP1	Protein NipSnap homolog 1	Member of the NipSnap protein family, potentially involved in vesicular transport.
Q9BPX5	ARPC5L	Actin-related protein 2/3 complex subunit 5-like protein	Actin-binding protein involved in Arp2/3 complex–mediated actin nucleation and cell migration.
Q9Y2T2	AP3M1	AP-3 complex subunit mu-1	Facilitates Golgi-derived vesicle budding and protein sorting to the endosomal/lysosomal system.
O94874	UFL1	E3 UFM1-protein ligase 1	UFM1 ligase involved in regulation of cell proliferation, proteasomal protein degradation, and responses to endoplasmic reticulum stress.
P08134	RHOC	Rho-related GTP-binding protein RhoC	Rho-family small GTPase acting as a molecular switch to regulate actin cytoskeleton organization, cell shape, adhesion, and motility.
Q8IWS0	PHF6	PHD finger protein 6	Nucleolar protein containing two PHD-type zinc finger domains, suggesting a role in transcriptional regulation.
Q9H2W6	MRPL46	39S ribosomal protein L46, mitochondrial	Mitochondrial ribosomal protein required for protein synthesis within mitochondria.
P52298	NCBP2	Nuclear cap-binding protein subunit 2	5′ cap-binding protein involved in mRNA processing and nuclear export.
P52594	AGFG1	Arf-GAP domain and FG repeats-containing protein 1	Nucleoporin-related protein required for nuclear export of Rev-dependent RNAs.
O15344	MID1	Midline-1	Likely involved in formation of multiprotein complexes that anchor microtubules.
P08237	PFKM	6-phosphofructokinase, muscle type	Catalyzes the phosphorylation of fructose-6-phosphate to fructose-1,6-bisphosphate in glycolysis.
P30049	ATP5D	ATP synthase subunit delta, mitochondrial	Component of the mitochondrial F_0F_1 ATP synthase complex.
P46977	STT3A	Dolichyl-diphosphooligosaccharide--protein glycosyltransferase subunit STT3A	Catalytic subunit of the oligosaccharyltransferase complex mediating N-linked glycosylation in the endoplasmic reticulum.
P51572	BCAP31	B-cell receptor-associated protein 31	Endoplasmic reticulum multi-pass membrane protein involved in ER-to-Golgi transport and caspase-8–mediated apoptosis.
Q5JTV8	TOR1AIP1	Torsin-1A-interacting protein 1	Inner nuclear membrane protein potentially involved in nuclear envelope–lamina attachment during cell division.
O95292	VAPB	Vesicle-associated membrane protein-associated protein B/C	Forms homo- and heterodimers with VAPA; interacts with VAMP1 and VAMP2 and participates in vesicle trafficking.
P46459	NSF	Vesicle-fusing ATPase	PDZ-domain–binding protein involved in intracellular protein transport, receptor recycling, and protein catabolism.
Q15102	PAFAH1B3	Platelet-activating factor acetylhydrolase IB subunit gamma	Platelet-activating factor acetylhydrolase catalytic subunit involved in PAF inactivation and brain development.
Q7Z4W1	DCXR	L-xylulose reductase	Homotetrameric enzyme catalyzing diacetyl and L-xylulose reductase reactions, involved in the uronate cycle and renal osmoregulation.
Q96DH6	MSI2	RNA-binding protein Musashi homolog 2	Transcriptional regulator controlling genes involved in development and cell cycle progression.
Q9H3P7	ACBD3	Golgi resident protein GCP60	Transcriptional regulator involved in development and cell cycle control. Also contributes to Golgi structure and function via interaction with giantin and participates in hormonal regulation of steroidogenesis.
Q9NTJ5	SACM1L	Phosphatidylinositide phosphatase SAC1	Phosphoinositide phosphatase that hydrolyzes PI3P, PI4P, and $PI(3,5)P_2$.
P61421	ATP6V0D1	V-type proton ATPase subunit d 1	Component of the vacuolar ATPase complex, mediating acidification of intracellular organelles required for protein sorting, receptor-mediated endocytosis, zymogen activation, and synaptic vesicle proton gradient generation.
Q96S97	MYADM	Myeloid-associated differentiation marker	Involved in negative regulation of actin filament polymerization and heterotypic cell–cell adhesion, and positive regulation of adhesion-dependent cell spreading.
Q9P0L0	VAPA	Vesicle-associated membrane protein-associated protein A	Functions in vesicle trafficking, membrane fusion, protein complex assembly, and cell motility.
P56537	EIF6	Eukaryotic translation initiation factor 6	Translation initiation factor that prevents association of the 40S and 60S ribosomal subunits.
Q6P5R6	RPL22L1	60S ribosomal protein L22-like 1	RNA-binding protein and structural constituent of the ribosome, involved in cytoplasmic translation.
Q8TAT6	NPLOC4	Nuclear protein localization protein 4 homolog	Ubiquitin-binding protein involved in negative regulation of RIG-I signaling and type I interferon production, and in proteolysis during protein catabolism.
Q9Y305	ACOT9	Acyl-coenzyme A thioesterase 9, mitochondrial	Mitochondrial acyl-CoA thioesterase of unknown function.
O00560	SDCBP	Syntenin-1	Links syndecan-mediated signaling to the cytoskeleton, affecting cytoskeletal–membrane organization, cell adhesion, protein trafficking, and transcription factor activation.
P18031	PTPN1	Tyrosine-protein phosphatase non-receptor type 1	Negative regulator of insulin signaling by dephosphorylating phosphotyrosine residues of the insulin receptor kinase. Dephosphorylates EGFR, JAK2, and TYK2, implicating this PTP in cell growth control and interferon responses.
B2RPK0	HMGB1L1	Putative high mobility group protein B1-like 1	Represents an evolving retropseudogene of the high-mobility group box 1 gene, involved in DNA binding and regulation of transcription.
Q9NX63	CHCHD3	Coiled-coil-helix-coiled-coil-helix domain-containing protein 3, mitochondrial	Inner mitochondrial membrane scaffold protein required for cristae integrity, ATP production, cell growth, and oxygen consumption.
Q9Y6A4	UPF0468	UPF0468 protein C16orf80	RNA-binding protein involved in regulation of feeding behavior, protein polyglutamylation, and control of ciliary beat frequency.
Q99961	SH3GL1	Endophilin-A2	Protein involved in endocytosis and cell cycle regulation.
Q9NRZ9	HELLS	Lymphoid-specific helicase	Lymphoid-specific RNA helicase involved in cellular proliferation and leukemogenesis.
O00308	WWP2	NEDD4-like E3 ubiquitin-protein ligase WWP2	Protein involved in ubiquitination containing four WW domains, regulating chondrogenesis and oncogenic signaling via Smad proteins and PTEN.
P61964	WDR5	WD repeat-containing protein 5	Scaffold protein that facilitates formation of heterotrimeric or multiprotein complexes involved in cell cycle progression, signal transduction, apoptosis, and gene regulation.
Q13045	FLII	Protein flightless-1 homolog	Actin-binding protein involved in cytoskeletal remodeling, cell migration, and regulation of transcriptional responses.
Q9Y450	HBS1L	HBS1-like protein	GTP-binding elongation factor–like protein involved in ribosome recycling and mRNA surveillance during translation.
P62487	POLR2G	DNA-directed RNA polymerase II subunit RPB7	Transcription initiation factor that stabilizes RNA polymerase during elongation.
P69849	NOMO3	Nodal modulator 3	Protein participating in the Nodal signaling pathway during vertebrate development.
Q9ULH0	KIDINS220	Kinase D-interacting substrate of 220 kDa	Scaffold protein mediating crosstalk between neurotrophin signaling and MAPK pathways.
P45974	USP5	Ubiquitin carboxyl-terminal hydrolase 5	Deubiquitinating enzyme that disassembles branched polyubiquitin chains via a sequential exo mechanism.
P98170	XIAP	Baculoviral IAP repeat-containing protein 4	Protein that binds TRAF1 and TRAF2 and inhibits apoptosis.
Q16891	IMMT	Mitochondrial inner membrane protein	RNA-binding protein involved in mitochondrial cristae formation.
Q99567	NUP88	Nuclear pore complex protein Nup88	Nuclear transport protein that regulates macromolecular trafficking between the nucleus and cytoplasm.
O43719	HTATSF1	HIV Tat-specific factor 1	Cofactor that couples HIV-1 Tat–dependent transcriptional elongation with RNA splicing.
P61457	PCBD1	Pterin-4-alpha-carbinolamine dehydratase	Dehydratase involved in tetrahydrobiopterin biosynthesis and cofactor for HNF1A-dependent transcription.

	Q9NNW5	WDR6	WD repeat-containing protein 6	Protein interacting with serine/threonine kinase 11 and involved in cell growth arrest.
	P11310	ACADM	Medium-chain specific acyl-CoA dehydrogenase, mitochondrial	Enzyme catalyzing the initial step of mitochondrial fatty acid β-oxidation.
	Q13873	BMPR2	Bone morphogenetic protein receptor type-2	Serine/threonine kinase receptor for BMP ligands involved in endochondral bone formation and embryogenesis.
	Q5H9R7	SAPS3	Serine/threonine-protein phosphatase 6 regulatory subunit 3	Regulatory subunit of protein phosphatase PP6 that modulates phosphatase activity.
	P29508	SERPINB3	Serpin B3	Cysteine protease inhibitor with protease-binding and virus receptor activity involved in autocrine and paracrine signaling.
	Q16658	FSCN1	Fascin	Actin-bundling protein required for formation of actin-based protrusions and regulation of cell migration, adhesion, and motility.
	Q14562	DHX8	ATP-dependent RNA helicase DHX8	ATP-dependent RNA helicase involved in releasing spliced mRNAs from spliceosomes prior to nuclear export.
	Q96RP9	GFM1	Elongation factor G, mitochondrial	Mitochondrial translation elongation factor required for normal mitochondrial function.
	Q7L576	CYFIP1	Cytoplasmic FMR1-interacting protein 1	Component of the WAVE regulatory complex regulating cytoskeletal dynamics and promoting actin polymerization.
	P19105	MYL12A	Myosin regulatory light chain 12A	Regulatory light chain of nonmuscle myosin II that controls cellular contractility.
	P49419	ALDH7A1	Alpha-aminoadipic semialdehyde dehydrogenase	Enzyme involved in detoxification of aldehydes generated by alcohol metabolism and lipid peroxidation.
	P13984	GTF2F2	General transcription factor IIF subunit 2	Transcriptional regulator involved in RNA polymerase II initiation and elongation.
	O14874	BCKDK	[3-methyl-2-oxobutanoate dehydrogenase [lipoamide]] kinase, mitochondrial	Kinase that regulates branched-chain amino acid catabolism by phosphorylating BCKDH.
	O14965	AURKA	Serine/threonine-protein kinase 6	Serine/threonine kinase that regulates mitosis, centrosome function, and spindle assembly.
	Q96HC4	PDLIM5	PDZ and LIM domain protein 5	Scaffold protein that anchors kinases to the Z-disk, regulating cardiomyocyte expansion and synaptic growth.
	Q14C86	GAPVD1	GTPase-activating protein and VPS9 domain-containing protein 1	Protein with GTPase-activating and guanine nucleotide exchange factor activities involved in protein transport.
	P33176	KIF5B	Kinesin-1 heavy chain	Microtubule motor protein involved in vesicle transport, protein localization, and natural killer cell–mediated cytotoxicity.
	P02671	FGA	Fibrinogen alpha chain	Alpha subunit of fibrinogen, proteolytically processed by thrombin during fibrin formation.
	O75663	TIPRL	TIP41-like protein	Inhibitory regulator of protein phosphatases PP2A, PP4, and PP6.
	Q14126	DSG2	Desmoglein-2	Calcium-binding transmembrane glycoprotein component of desmosomes involved in cell–cell adhesion.
	P23458	JAK1	Tyrosine-protein kinase JAK1	Tyrosine kinase associated with cytokine receptors that mediates JAK/STAT signaling and regulates inflammation and cancer progression.
	P49959	MRE11A	Double-strand break repair protein MRE11A	Nuclear protein involved in homologous recombination, telomere maintenance, and DNA double-strand break repair.
	Q8TEQ6	GEMIN5	Gem-associated protein 5	Component of the SMN complex involved in snRNP biogenesis and RNA splicing.
Common proteins	Q5XKP0	QIL1	Protein QIL1	Inner mitochondrial membrane protein of the MICOS complex involved in cristae organization.
	Q9BZL1	UBL5	Ubiquitin-like protein 5	Ubiquitin-like protein involved in cell proliferation, sister chromatid cohesion, and spliceosome integrity.
	Q08209	PPP3CA	Serine/threonine-protein phosphatase 2B catalytic subunit alpha isoform	Protein involved in calcineurin–NFAT signaling, angiotensin signaling regulation, and serine dephosphorylation.
	Q9H3N1	TMX1	Thioredoxin-related transmembrane protein 1	Endoplasmic reticulum redox protein involved in protein folding and redox homeostasis.
	Q9NZL9	MAT2B	Methionine adenosyltransferase 2 subunit beta	Methionine adenosyltransferase family enzyme that synthesizes S-adenosylmethionine from methionine and ATP.
	O75844	ZMPSTE24	CAAX prenyl protease 1 homolog	Zinc metalloproteinase required for post-translational processing of prelamin A into mature lamin A.
	Q9UL25	RAB21	Ras-related protein Rab-21	Protein involved in integrin trafficking via interaction with integrin alpha tails.
	P59998	ARPC4	Actin-related protein 2/3 complex subunit 4	Actin-related protein complex subunit required for actin nucleation and high-affinity binding to F-actin.
	Q00688	FKBP3	Peptidyl-prolyl cis-trans isomerase FKBP3	Protein involved in immunoregulation and cellular protein folding and trafficking.
	P63092	GNAS	Guanine nucleotide-binding protein G(s) subunit alpha isoforms short	Stimulatory G protein alpha subunit (Gαs) that links GPCR activation to adenylyl cyclase and cAMP production.
	P04080	CSTB	Cystatin-B	Cysteine protease inhibitor that protects against protease-mediated damage.
	P27695	APEX1	DNA-(apurinic or apyrimidinic site) lyase	APEX endonuclease involved in base excision DNA repair with apurinic/apyrimidinic activity.
	P14324	FDPS	Farnesyl pyrophosphate synthase	Enzyme that synthesizes geranyl pyrophosphate and farnesyl pyrophosphate from isoprenoid precursors.
	Q13547	HDAC1	Histone deacetylase 1	Component of histone acetylation and deacetylation complexes involved in regulation of gene expression.
	P50213	IDH3A	Isocitrate dehydrogenase [NAD] subunit alpha, mitochondrial	Enzyme that catalyzes the oxidative decarboxylation of isocitrate to 2-oxoglutarate.
	Q15417	CNN3	Calponin-3	Thin filament–associated protein involved in regulation of smooth muscle contraction.
	P62633	CNBP	Cellular nucleic acid-binding protein	Protein involved in cap-independent translation of ornithine decarboxylase mRNA and sterol-mediated transcriptional regulation.
	Q14677	CLINT1	Clathrin interactor 1	Protein involved in clathrin-mediated endocytosis and vesicular trafficking.

Supplementary Table S2. Cannabinoid and terpene composition of the cannabis-derived extracts. CE1 was donated by Aunt Zelda's (California, USA), and CE2 and CE3 were donated by Curativa Group (Colombia). Concentrations are expressed in mg/g. BDL = Below detection limit.

**CANNABIS EXTRACT 1 (CE1)**	
CANNABINOID	CONCENTRATION
THCA	BDL
**THC**	**150.1**
THCV	2.1
CBD	6.0
CBDA	BDL
CBG	24.1
CBN	5.5
CBC	6
TERPENE	CONCENTRATION
α-Bisabolol	BDL
Camphene	0.014
3-Carene	0.003
β-Caryophyllene	2.074
Caryophyllene Oxide	0.001
p-Cymene	BDL
Geraniol	0.002
Guaiol	0.074
α-Humulene	0.001
Isopulegol	0.004
D-Limonene	0.914
Linalool	0.906
β-Myrcene	0.841
Nerolidol 1	0.005
Nerolidol 2	1.331
Ocimene	0.010
α-Pinene	0.939
β-Pinene	0.002
α-Terpinene	0.001
γ-Terpinene	0.003
Terpinolene	0.003

**CANNABIS EXTRACT 2 (CE2)**	
CANNABINOID	CONCENTRATION
THCA	3.5
**THC**	**551.3**
THCV	BDL
CBD	BDL
CBDA	BDL
CBG	3.6
CBN	BDL
CBC	BDL
TERPENE	CONCENTRATION
α-Bisabolol	0.177
Camphene	BDL
3-Carene	BDL
β-Caryophyllene	1.948
Caryophyllene Oxide	0.032
p-Cymene	0.178
Geraniol	BDL
Guaiol	BDL
α-Humulene	0.557
Isopulegol	0.023
D-Limonene	BDL
Linalool	0.620
β-Myrcene	0.025
Nerolidol 1	0.357
Nerolidol 2	0.081
Ocimene	0.049
α-Pinene	0.015
β-Pinene	0.317
α-Terpinene	0.013
γ-Terpinene	0.013
Terpinolene	0.017

**CANNABIS EXTRACT 3 (CE2)**	
CANNABINOID	CONCENTRATION
THCA	BDL
**THC**	**73.6**
THCV	1.0
**CBD**	**84.8**
CBDA	BDL
CBG	13.0
CBN	3.7
CBC	5.8
TERPENE	CONCENTRATION
α-Bisabolol	BDL
Camphene	0.010
3-Carene	0.002
β-Caryophyllene	2.087
Caryophyllene Oxide	0.002
p-Cymene	BDL
Geraniol	0.001
Guaiol	0.041
α-Humulene	0.001
Isopulegol	0.002
D-Limonene	0.860
Linalool	0.901
β-Myrcene	0.790
Nerolidol 1	0.002
Nerolidol 2	1.270
Ocimene	0.004
α-Pinene	0.793
β-Pinene	0.002
α-Terpinene	0.002
γ-Terpinene	0.002
Terpinolene	0.003
